# Arabidopsis WALL-ASSOCIATED KINASES are not required for oligogalacturonide-induced signaling and immunity

**DOI:** 10.1093/plcell/koae317

**Published:** 2024-12-12

**Authors:** Laura Herold, Jana Ordon, Chenlei Hua, Bruce D Kohorn, Thorsten Nürnberger, Thomas A DeFalco, Cyril Zipfel

**Affiliations:** Institute of Plant and Microbial Biology and Zürich-Basel Plant Science Center, University of Zürich, Zürich 8008, Switzerland; Institute of Plant and Microbial Biology and Zürich-Basel Plant Science Center, University of Zürich, Zürich 8008, Switzerland; Center of Plant Molecular Biology (ZMBP), University of Tübingen, Tübingen 72076, Germany; Department of Biology, Bowdoin College, Brunswick, ME 04011, USA; Center of Plant Molecular Biology (ZMBP), University of Tübingen, Tübingen 72076, Germany; Institute of Plant and Microbial Biology and Zürich-Basel Plant Science Center, University of Zürich, Zürich 8008, Switzerland; Institute of Plant and Microbial Biology and Zürich-Basel Plant Science Center, University of Zürich, Zürich 8008, Switzerland; The Sainsbury Laboratory, University of East Anglia, Norwich Research Park, Norwich NR4 7UH, UK

## Abstract

Carbohydrate-based cell wall signaling impacts plant growth, development, and stress responses; however, how cell wall signals are perceived and transduced remains poorly understood. Several cell wall breakdown products have been described as typical damage-associated molecular patterns that activate plant immunity, including pectin-derived oligogalacturonides (OGs). Receptor kinases of the WALL-ASSOCIATED KINASE (WAK) family bind pectin and OGs and were previously proposed as OG receptors. However, unambiguous genetic evidence for the role of WAKs in OG responses is lacking. Here, we investigated the role of Arabidopsis (*Arabidopsis thaliana*) WAKs in OG perception using a clustered regularly interspaced short palindromic repeats mutant in which all 5 *WAK* genes were deleted. Using a combination of immune assays for early and late pattern-triggered immunity, we show that WAKs are dispensable for OG-induced signaling and immunity, indicating that they are not bona fide OG receptors.

## Teaser

Analysis of Arabidopsis mutants shows that WALL-ASSOCIATED KINASES are dispensable for oligogalacturonide (OG)-induced signaling and immunity, questioning their proposed role as OG receptors.

## Introduction

Plants are exposed to myriad potential pests and pathogens, against which they have evolved sophisticated defense mechanisms. The plant cell wall acts as the initial physical barrier against invasion, and alterations in this structure intricately interact with the plant immune system (Dora et al. 2022; Wolf 2022).

The plant cell wall is composed of cellulose, hemicellulose, pectin, polyphenolic lignin, and a series of structural and enzymatically active proteins (Wolf 2022; Cosgrove 2023). Cell wall polysaccharides serve as extracellular sources for the generation of damage-associated molecular patterns (DAMPs) that are thought to be released upon mechanical damage or pathogen infection (Pontiggia et al. 2020). Several such carbohydrate DAMPs have been previously described, including cellulose-derived cellobiose and cellotriose, mixed linked glucans, and pectin-derived oligogalacturonides (OGs) (Bacete et al. 2018; Oelmüller et al. 2023). OGs represent the best studied pectin-derived cell wall breakdown products and can be generated from both methylesterified and demethylesterified pectins. The best studied OGs are, however, those that derived from demethylesterified pectins. OGs with a degree of polymerization 10 to 15 (OG_10–15_) have been shown to elicit canonical pattern-triggered immunity (PTI) signaling and confer plant protection against a range of pathogens (Bishop et al. 1981; Hahn et al. 1981; Ridley et al. 2001; De Lorenzo et al. 2011). More recently, shorter OGs such as trimers (GalA_3_/OG_3_) and tetramers have also been shown to trigger immune responses and defense (Davidsson et al. 2017; Liu et al. 2023; Xiao et al. 2024).

In the model plant *Arabidopsis thaliana* (hereafter, Arabidopsis), demethylated pectin was shown to directly bind the extracellular domain (ECD) of several WALL-ASSOCIATED KINASES (WAKs) (Decreux and Messiaen 2005; Decreux et al. 2006; Kohorn et al. 2009; Liu et al. 2023). WAKs belong to a large family of receptor kinases (RKs) comprising 5 WAKs and at least 21 WAK-likes (WAKLs) (Verica and He 2002) that are characterized by epidermal growth factor (EGF)-like domains and a galacturonan-binding domain in their ECD (He et al. 1996; Verica and He 2002; Kohorn et al. 2012; Stephens et al. 2022). WAK1 was the first identified RK physically linking the plasma membrane to the cell wall, as isolation from fractions of proteolytically digested cell walls indicated a strong interaction of WAK1 with the cell wall and native pectin in vivo (He et al. 1996; Anderson et al. 2001; Wagner and Kohorn 2001). Further experiments suggested WAKs and their association with cell wall pectin are involved in cell expansion (Kohorn et al. 2006) and potentially the response to pathogens (He et al. 1998; Kohorn et al. 2012). Later, WAK1 was also shown to bind pectin and OG_9–15_ with a high affinity in vitro (Decreux and Messiaen 2005; Decreux et al. 2006; Kohorn et al. 2009), with the WAK1-ECD preferentially interacting with de-esterified pectin through a binding site formed by cationic amino acids (Decreux and Messiaen 2005; Decreux et al. 2006).

A chimeric approach using the ECD of WAK1 fused to the intracellular domain of the leucine-rich repeat RK ELONGATION FACTOR-TU RECEPTOR (EFR) served as evidence for a proposed role for WAK1 in OG perception (Brutus et al. 2010). Although OG treatment of WAK1-EFR chimera-expressing plants induced an EFR cytosolic domain-mediated defense response, critical genetic evidence that WAKs are bona fide OG receptors is still lacking. Direct genetics of *WAK*s was previously hindered by the genetic clustering of the *WAK* family in Arabidopsis, the assumption that *wak1* null mutants were lethal, and expected functional redundancy among the 5 members of the WAK family (He et al. 1999; Brutus et al. 2010; Kohorn and Kohorn 2012). Recently, however, a clustered regularly interspaced short palindromic repeat (CRISPR) deletion mutant for most of the chromosomal cluster carrying the 5 Arabidopsis *WAK* genes, *wakΔ*, was generated. This mutant was shown to be less sensitive to the bacterial flagellin-derived epitope flg22, chitin, and OGs in terms of reactive oxygen species (ROS) production (Kohorn et al. 2021), suggesting that WAKs may generally regulate immune receptor complexes, rather than function specifically as OG receptors (Wang et al. 2020; Zhang et al. 2020). WAKs were also recently shown to be genetically required for GalA_3_/OG_3_-induced expression of the salicylic acid (SA) marker gene *PATHOGENESIS-RELATED 1 (PR1)* (Liu et al. 2023).

In this work, we directly investigated the genetic involvement of WAKs in OG-induced signaling in Arabidopsis. We generated a deletion of the entire *WAK1–5* region (*wakΔ2*) and tested this mutant for OG-induced responses. Surprisingly, we found that *wakΔ2* retained full responsiveness to OGs, as measured by both early and late outputs of immune signaling. In addition, *wakΔ2* plants were not affected in OG-induced resistance against both bacterial and fungal pathogens. Furthermore, we tested the genetic involvement of WAKs in response to flg22 and could observe that flg22-induced responses are not affected in the *wakΔ2* mutant. Together, our data indicate that WAKs are not genetically required for OG perception and ensuing immune signaling in Arabidopsis.

## Results

### Generation of the *wakΔ2* mutant

**Figure 1.**
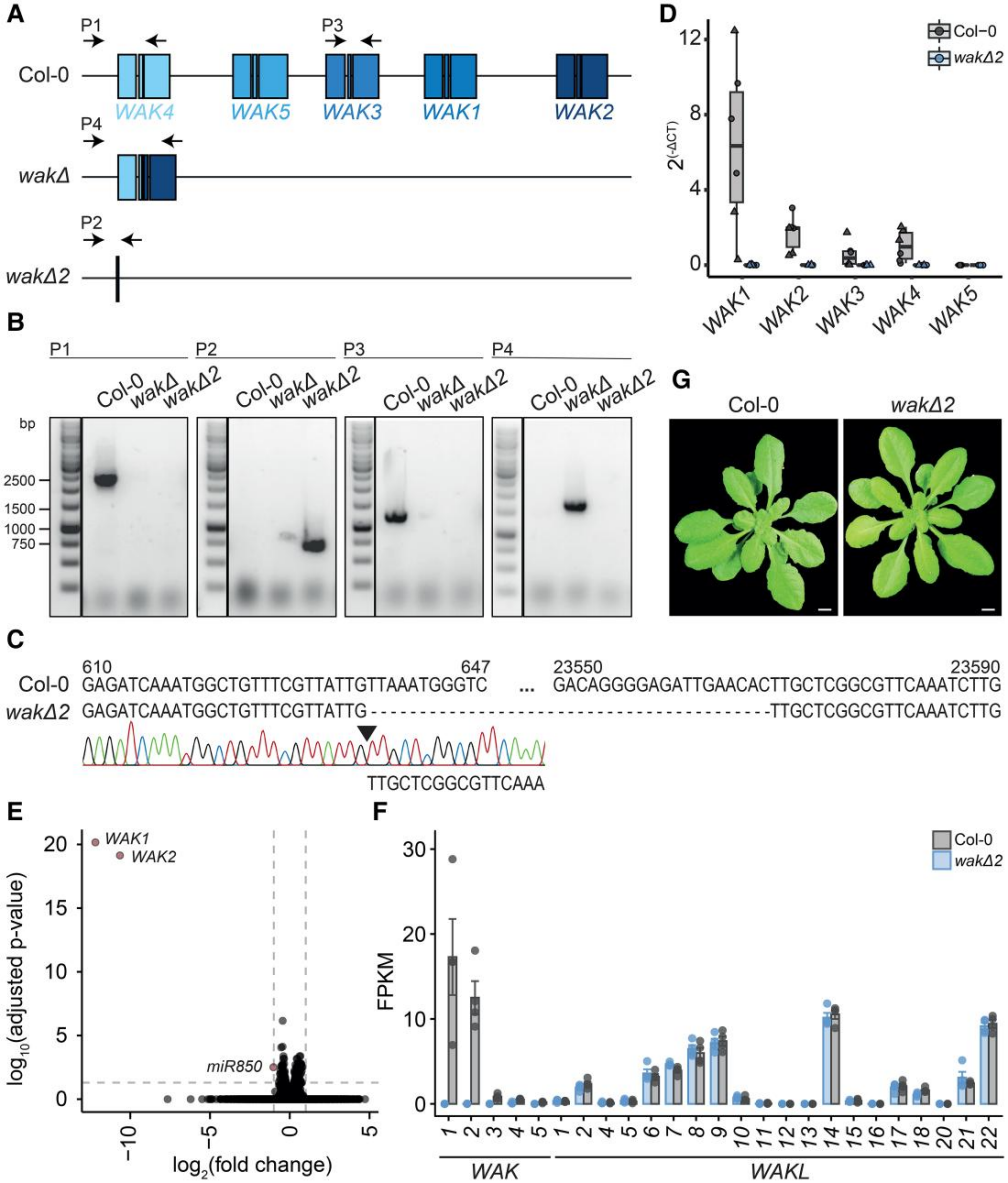
*WakΔ2* is a full WAK deletion mutant. **A)** Schematic representation of the genomic arrangement of *WAK1–5* in Arabidopsis. The middle cartoon shows the genomic deletion in *wakΔ* and the consequent fusion of WAK4 and WAK2. The lower cartoon shows the genomic region of the *WAK* cluster in *wakΔ2*. Black arrows indicate primer pairs (P) used in B. **B)** Genotyping gel of Col-0, *wakΔ*, and *wakΔ2*. Ethidium bromide-stained PCR products for parts indicated in A on agarose gel. P1–P4 refer to the primer pairs shown in A. bp, base pair. **C)** Sequencing results from the *wakΔ2* mutant aligned against the 5′UTR of *WAK4* and part of the third exon of *WAK2* of Col-0. **D)** Transcript levels of *WAK1–5* in Col-0 and *wakΔ2* determined by RT-qPCR. RNA was extracted from 14-d-old Arabidopsis seedlings grown in liquid culture. Transcripts were normalized to the house-keeping gene *UBOX*. Shapes indicate different biological replicates harvested at different times. This experiment was performed 2 times. CT, cycle threshold. **E)** Overall changes in Col-0 vs. *wakΔ2* transcriptomes in liquid-grown seedlings. **F)***WAK* and *WAK-like* transcript levels in Col-0 vs. *wakΔ2*. FPKM, fragments per kilobase of transcripts per million mapped reads. **G)** Photos of 4-wk-old Arabidopsis plants grown on soil. Images were digitally extracted for comparison. Scale bar = 1 cm.

The Arabidopsis genome has 5 *WAK* genes located in a cluster on chromosome 1 (Fig. 1A). Recently, a partial deletion mutant was published, *wakΔ,* which lacks most of the cluster; however, this mutant still potentially expresses a fusion protein of the N-terminal region of WAK4 and the C-terminal region of WAK2 (Fig. 1A) (Kohorn et al. 2021). While the *wakΔ* mutant showed partially impaired flg22, chitin, and OG responsiveness, it suffered from the presence of this potential WAK4-WAK2 fusion protein. Therefore, to explore whether WAKs are genetically required for OG-induced responses, we deleted the remaining *WAK4-WAK2* fusion in the Arabidopsis Col-0 *wakΔ* mutant by using CRISPR/Cas9 to generate the *wakΔ2* mutant, in which all *WAK* genes are absent (Fig. 1, A to D). We validated the cluster deletion by Sanger sequencing of the borders of the *WAK* cluster (Fig. 1, A to C). Lack of *WAK1–5* expression in 2-wk-old *wakΔ2* seedlings was confirmed using RT-qPCR (Fig. 1D). We also performed whole-genome RNA-seq comparing Col-0 and *wakΔ2* seedlings (Fig. 1, E and F). The analysis first showed that, in the *wakΔ2* mutant, no reads were detected for *WAKs* except for *WAK4*, for which ∼600 nt of the 5′ UTR are still encoded, thus independently confirming that *wakΔ2* is a true loss-of-function mutant for the 5 Arabidopsis *WAK* genes. Surprisingly, only 3 genes were significantly differentially expressed between Col-0 and *wakΔ2*: *WAK1*, *WAK2*, and *miR850* (Fig. 1E), in line with the deletion of these genes and the very low expression of *WAK3*, *4*, and *5* under these conditions (Fig. 1D). These results are consistent with previously published RNA-seq data of the *wakΔ* mutant (Kohorn et al. 2021), showing no major transcriptional changes except for the deleted *WAKs*. Finally, the expression of *WAKLs* was unaltered in the *wakΔ2* mutant (Fig. 1F), indicating that there is no compensatory mechanism due to the loss of *WAKs*. In agreement with the previously published *WAK* deletion mutants (Kohorn et al. 2021; Liu et al. 2023), *wakΔ2* displayed no obvious growth phenotype when grown on soil (Fig. 1G).

### OG-induced immune signaling does not require the WAK family

Given that WAKs are proposed as receptors perceiving OGs (Brutus et al. 2010), we investigated whether the cluster encoding *WAK1–5* is genetically required for OG-induced immune responses using the *wakΔ2* mutant. Previously, OG_10–15_ were shown to induce various immune responses in Arabidopsis including extracellular ROS production, mitogen-activated protein kinase (MAPK) activation, marker gene expression, ethylene production, callose deposition, seedling growth inhibition, and resistance against pathogens (Denoux et al. 2008; Davidsson et al. 2017; Gravino et al. 2017; Bjornson et al. 2021). Single and higher-order mutants of closely related receptors that perceive peptides (e.g. PEPR1/2 and HAE/HSL2), glycans (e.g. LYK4/LYK5), or small molecules (e.g. P2K1/P2K2) are typically completely insensitive to treatment with the corresponding ligand (Gómez-Gómez and Boller 2000; Chinchilla et al. 2006; Zipfel et al. 2006; Miya et al. 2007; Cho et al. 2008; Yamaguchi et al. 2010; Cao et al. 2014; Choi et al. 2014; Ranf et al. 2015; Pham et al. 2020; Rhodes et al. 2021). We therefore hypothesized that loss-of-function mutants of a *bona fide* OG receptor should not be able to induce OG-induced responses.

**Figure 2.**
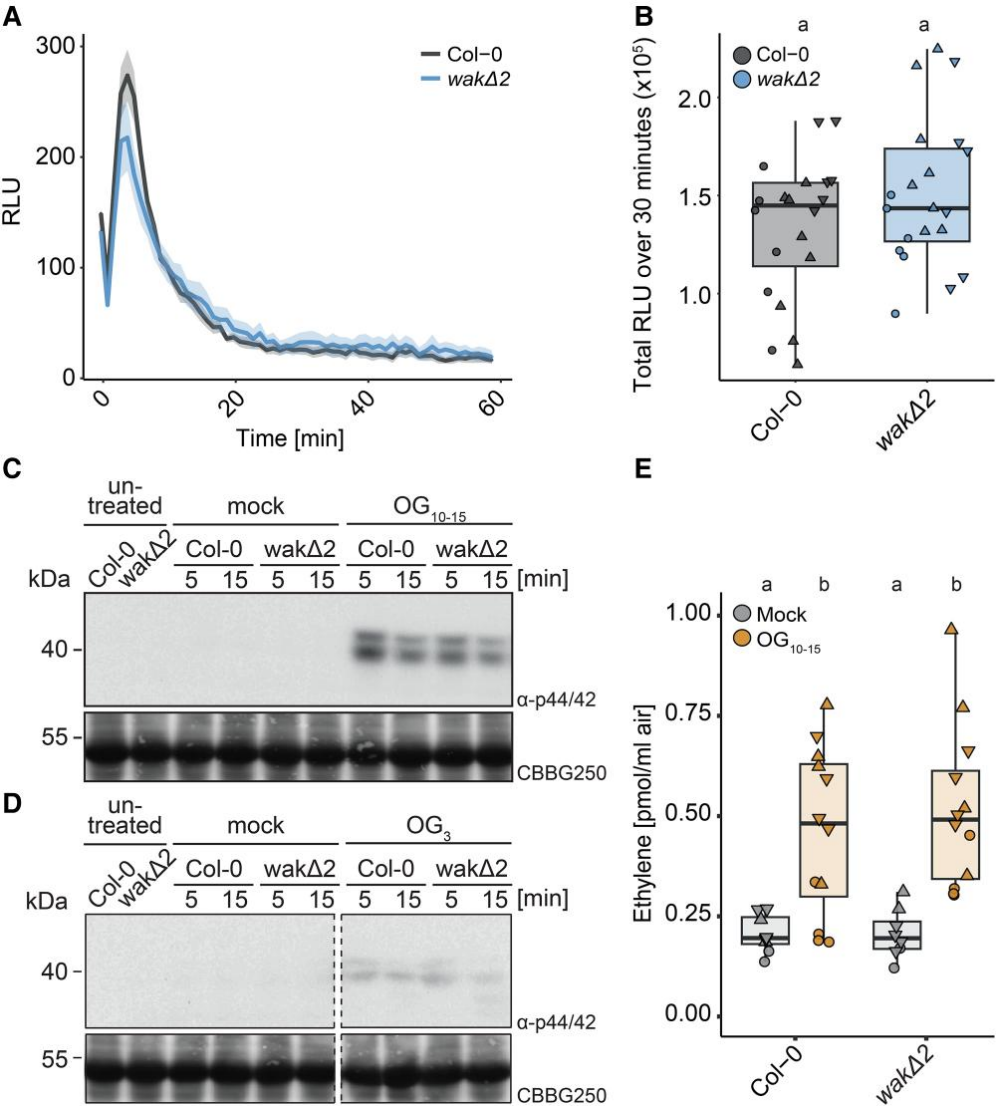
WAKs are not required for OG-induced early immune responses. **A** and **B)** ROS production in response to OG_10–15_ in leaf discs of 3- to 4-wk-old Arabidopsis plants. 100 µg/mL to 1 mg/mL of OG_10–15_ were used as concentration dependent of the experiment. RLU, relative luminescent units. **A)** Representative graph of the kinetics of 1 replicate (*n* = 12 leaf discs of 6 plants). 1 mg/mL OG_10–15_ was used as concentration. Mean ± SE are plotted. **B)** Values are means of total photon counts over 30 min. Data from 3 independent experiments are shown (*n* = 6 to 8 plants dependent on the experiment). Shapes indicate different replicates. Statistical test: Kruskal–Wallis test (*P* = 0.3302). Groups with like letter designations are not statistically different. **C** and **D)** MAPK activation assay with 2-wk-old seedlings in response to 100 µg/mL OG_10–15_ (C), 100 µg/mL OG_3_ (D) or mock (C, D). Samples were collected 0, 5, and 15 min after elicitation as indicated. Blots were probed with α-p44/42. Coomassie brilliant blue (CBB) was used as loading control. **E)** Ethylene accumulation after treatment with 100 µg/mL OG_10–15_ or water as control in 4- to 6-wk-old Arabidopsis leaves. Individual data points represent measured plants from 3 replicates indicated by different shapes performed at different days. Equal letters at the top of the panel indicate *P* > 0.05, Kruskal–Wallis test (*P*-value = 0.0001058) with Dunn’s post hoc multiple comparison test. These experiments were performed 3 times at different times. **B)** and **C)** Boxplots show median with 0.25 and 0.75 quartiles. Whiskers represent 1.5 and -1.5 times the interquartile range.

To investigate whether early immune signaling induced by OGs is dependent on WAKs, we measured extracellular ROS production in leaves of 3- to 4-wk-old Arabidopsis plants. Surprisingly and in contrast to previous results (Kohorn et al. 2021), OG_10–15_-induced ROS production was unaltered in leaves of *wakΔ2* in comparison with Col-0 grown under short-day conditions (10 h of light) (Fig. 2, A and B). In addition to ROS, OGs induce rapid and transient MAPK phosphorylation (Gravino et al. 2017). To determine whether OG-induced MAPK activation is affected in the *wakΔ2* mutant, MAPK phosphorylation was determined in Arabidopsis seedlings 5 and 15 min after elicitor treatment by western blot analysis using a commercial phosphorylation site-specific antibody. As with ROS production, OG_10–15_-triggered MAPK phosphorylation was unaltered in *wakΔ2* mutants (Fig. 2C). In addition to OG_10–15_, OG_3_ (GalA_3_) was previously shown to trigger MAPK phosphorylation and WAKs have been shown to be required for OG_3_-induced *PR1* expression (Davidsson et al. 2017; Liu et al. 2023). We therefore additionally investigated if OG_3_-induced MAPK phosphorylation is dependent on WAKs. OG_3_-induced MAPK phosphorylation is comparatively weak but was still induced in *wakΔ2* mutant plants (Fig. 2D). OGs were also previously shown to induce synthesis of ethylene in Arabidopsis seedlings (Ferrari et al. 2008; Brutus et al. 2010; Gravino et al. 2015). In line with other early induced PTI pathways, OG_10–15_-induced ethylene production in leaves was not compromised in *wakΔ2* mutants (Fig. 2E). Taken together, these results indicate that *WAK1–5* are not required for OG-induced early immune outputs and thus for the signaling initiation of OG-induced responses.

**Figure 3.**
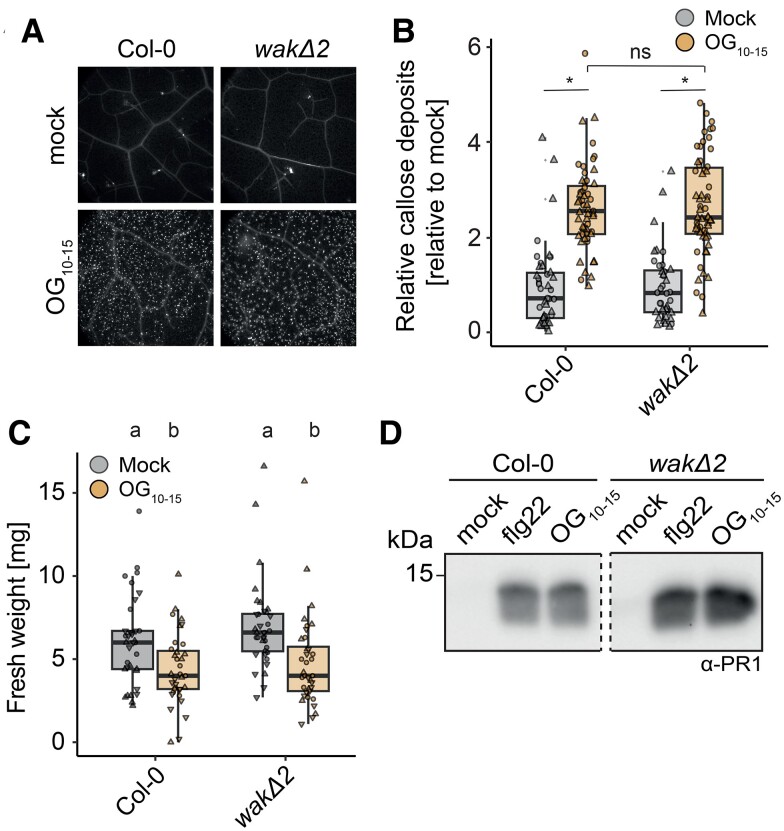
WAKs are not required for OG-induced late immune responses. **A** and **B)** Callose deposition in response to 100 µg/mL OG_10–15_ or water 24 h after infiltration into leaves of 3- to 4-wk-old Arabidopsis plants. *n* = 16 to 32 leaf discs from 4 different plants were taken per independent experiment. The experiment was performed 2 times with similar results. **A)** Representative images of OG-induced callose deposition in the presented genotypes stained with aniline blue. **B)** Relative callose deposits induced by OGs and water infiltration. Individual data points for each leaf disc are shown, and different shapes indicate individual experiments. Asterisks indicate statistical difference from mock treatment within 1 genotype (*P* < 0.05). Statistical test: Kruskal–Wallis test (*P*-value <2.2 × 10^−16^) with Dunn’s host hoc multiple comparison test. **C)** Fresh weight of 2-wk-old seedlings grown in liquid media for 10 d in the presence of 200 µg/mL OG_10–15_ or in the absence of neither (mock). Individual values for each plant and experiment (*n* = 12 to 14 seedlings per experiment) are shown, and different shapes indicate the different 3 replicates. Equal letters at the top of the panel indicate *P* > 0.05, Kruskal–Wallis test (*P*-value = 1.565 × 10^−5^) with Dunn’s post hoc multiple comparison test. Groups with like letter designations are not statistically different. The experiment was repeated 3 times with similar results. **D)** PR1 accumulation assessed by immunoblotting with PR1 antibodies. Leaves from 3-wk-old Arabidopsis plants were infiltrated with water (mock), 1 µM flg22, or 100 µg/mL OG_10–15_ and harvested after 24 h. The experiment was repeated 3 times with similar results. **B)** and **C)** Boxplots show median with 0.25 and 0.75 quartiles. Whiskers represent 1.5 and -1.5 times the interquartile range.

Aside from rapid signaling, PTI additionally involves longer-term responses such as callose deposition (Beffa et al. 1996; Luna et al. 2011; Wang et al. 2021). To investigate the requirement of WAKs at later stages of OG-induced responses, OG_10–15_-induced callose deposition was measured in leaf discs of 4- to 5-wk-old Col-0 and *wakΔ2* plants 24 h after infiltration of either water or 100 µg/mL OG_10–15_. OG_10–15_ induced callose deposition in both Col-0 and *wakΔ2* leaf tissues (Fig. 3, A and B). As is true of many elicitors, both OG_3_ and long OGs can inhibit plant growth (Davidsson et al. 2017). Arabidopsis seedlings grown in the presence of OG_10–15_ showed a significant growth inhibition in comparison with mock-treated seedlings; however, no difference could be observed between Col-0 and the *wakΔ2* mutant (Fig. 3C). Additionally, when grown in mock conditions, no growth difference could be observed between Col-0 and *wakΔ2* seedlings (Fig. 3C). Another long-term measurement of plant immune signaling is the production of SA and ensuing signaling, which can be inferred through the accumulation of the PR1 marker protein by immunoblotting (Tsuda et al. 2009; Zhang and Li 2019; Bender et al. 2021). OG_10–15_ and flg22 induced robust PR1 accumulation 24 h after elicitor infiltration into leaves of 3-wk-old Col-0 plants. Both flg22- and OG_10–15_-induced PR1 accumulation was not affected in *wakΔ2* plants (Fig. 3D).

Collectively, these results indicate that WAKs are not required for OG-induced immune signaling.

### WAKs are not required for OG-induced immunity

**Figure 4.**
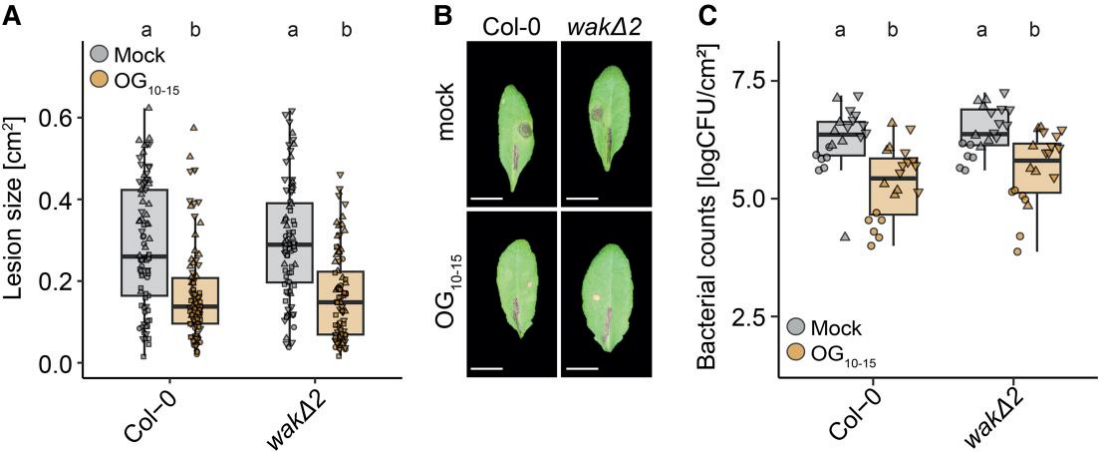
OG-induced immunity is not affected in wakΔ2. **A** and **B)** OG-induced resistance against *B. cinerea*. Four- to five-wk-old Col-0 or *wakΔ2* plants were infiltrated with water or 100 µg/mL OG_10–15_ 24 h prior drop inoculation with *B. cinerea* strain BMM spores (5 µL; 5 × 10^5^ spores/mL). Lesion areas were measured 48 h post-inoculation. The experiment was performed 4 times. **A)** Quantification of lesion sizes. Individual values for each leaf and experiment (*n* = 18 to 24 per experiment) are shown. Different shapes indicate different experiments. Equal letters at the top of the panel indicate *P* > 0.05, Kruskal–Wallis test (*P*-value = 8.572 × 10^−14^) with Dunn’s multiple comparison post hoc test. Groups with like letter designations are not statistically different. **B)** Representative images of OG-induced immunity in the different genotypes. Images were taken 48 h post-inoculation. Images were digitally extracted for comparison. Scale bar = 1 cm. **C)** OG-induced resistance against *P. syringae* pv tomato DC3000. Four- to five-wk-old plants were pretreated with water or 100 µg/mL OG_10–15_ for 24 h before infiltration with *P. syringae*. 48 h after *P. syringae* infiltration, bacteria were extracted and plated. Individual data points from the 3 pooled experiments (*n* = 6 per experiment, total = 18) are shown with different shapes per experiment. Equal letters at the top of the panel indicate *P* > 0.05, 2-way ANOVA with Tukey’s post hoc test. Groups with like letter designations are not statistically different. The experiment was performed 3 times. CFU, colony forming units. **A)** and **C)** Boxplots show median with 0.25 and 0.75 quartiles. Whiskers represent 1.5 and -1.5 times the interquartile range.

OGs have been shown to induce protection against the necrotrophic fungus *Botrytis cinerea*, the necrotrophic bacterium *Pectobacterium carotovorum*, and the hemibiotrophic bacterium *Pseudomonas syringae* (Davidsson et al. 2017; Gravino et al. 2017; Howlader et al. 2020). To investigate whether WAKs are required for OG-induced immunity, we drop-inoculated Arabidopsis Col-0 and *wakΔ2* leaves with *B. cinerea* conidia 24 h after infiltration with water or 100 µg/mL OG_10–15_. Disease lesions on leaves were measured 48 h post-inoculation. Plants pretreated with water showed significantly larger lesion sizes in both Col-0 and *wakΔ2* than plants that were pretreated with OG_10–15_ (Fig. 4, A and B). OG-induced protection against *B. cinerea* was not affected in *wakΔ2* plants in comparison with wild-type plants. OG-induced protection against *P. syringae* was similarly unaltered in leaves of *wakΔ2* in comparison with Col-0 (Fig. 4C). Overall, these results indicate that WAKs are not required for OG-induced immunity against these necrotrophic or hemibiotrophic pathogens.

### WAKs do not play a significant role in immune signaling triggered by other elicitors

**Figure 5.**
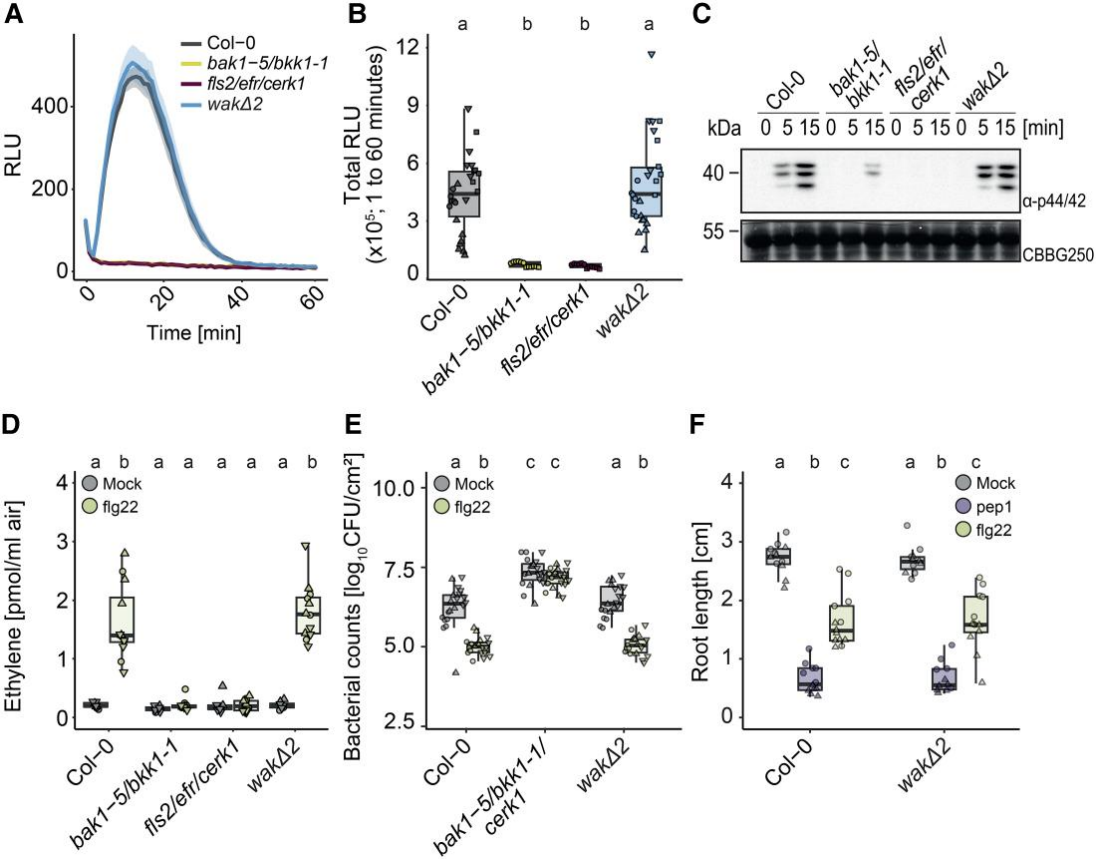
flg22-induced immunity is not affected by the loss of WAK1–5. **A** and **B)** ROS production in leaf discs of 3- to 4-wk-old plants using 100 nm flg22 in Col-0, *bak1-5/bkk1-1*, *fls2/efr/cerk1*, and *wakΔ2*. The experiment was repeated at least 3 times (A and B). Mean ± SEs are plotted. RLU, relative luminescent units. **A)** Kinetics of 3 representative independent replicates over 40 to 60 min. **B)** Values are means of total photon counts over 60 min as stated in the graph. Individual data points show ROS production in individual plants (*n* = 6 to 8 plants with each 2 leaf discs). Kruskal–Wallis test (*P*-value = 1.233 × 10^−10^) with Dunn’s post hoc test. Groups with like letter designations are not statistically different. **C)** MAPK activation assay with 2-wk-old seedlings in response to 1 µM flg22. Samples were collected 0, 5, and 15 min after elicitation as indicated. Blot was probed with α-p44/42. CBB was used as loading control. **D)** Ethylene accumulation after treatment with 1 µM flg22 or water as control in 4- to 6-wk-old Arabidopsis. Three pooled replicates with individual data points for each leaf are shown. Equal letters at the top of the panel indicate *P* > 0.05, Kruskal–Wallis test (*P*-value = 3.907 × 10^−9^) with Dunn’s post hoc multiple comparison test. **E)** OG-induced resistance against *P. syringae* pv tomato DC3000. Four- to five-wk-old plants were pretreated with water or 1 µM flg22 for 24 h before infiltration with *P. syringae*. 48 h after *P. syringae* infiltration, bacteria were extracted and plated. Individual data points from the 3 pooled experiments (*n* = 6 per experiment) are shown. Equal letters at the top of the panel indicate *P* > 0.05, Kruskal–Wallis test (*P*-value <2.2 × 10^−16^) with Dunn’s post hoc multiple comparison test. Groups with like letter designations are not statistically different. CFU, colony forming units. **F)** Primary root length of 9-d-old Col-0 and *wakΔ2*. Plants were grown on ½ MS plates for 5 d and then transferred to liquid MS +1% sucrose containing mock, 10 nm pep1, or 100 nm flg22. Root growth was determined after 4 d in liquid culture. Six plants were measured per experiment. Values correspond to length of each root in cm. Equal letters at the top of the panel indicate *P* > 0.05, 2-way ANOVA with Tukey’s post hoc test. All experiments were performed 3 times with similar results; only primary root length was only measured twice. **B)**, **D)**, **E)**, and **F)** Boxplots show median with 0.25 and 0.75 quartiles. Whiskers represent 1.5 and -1.5 times the interquartile range.

Aside from their role as potential OG receptors, WAKs were recently reported to function in immune signaling induced by bacterial flagellin in tomato (*Solanum lycopersicum*) and fungal chitin in cotton (*Gossypium hirsutum*) (Wang et al. 2020; Zhang et al. 2020). While in tomato only some flagellin-induced responses involved WAKs, e.g. callose deposition and antibacterial immunity, *Gh*WAK7A was broadly required for full responsiveness to fungal chitin but not to OGs in cotton (Wang et al. 2020; Zhang et al. 2020). In line with those observations, the Arabidopsis *wakΔ* mutant showed a reduction in ROS production induced by flg22, chitin, and OGs (Kohorn et al. 2021). Intrigued by these findings, we also tested whether flg22-induced responses are affected by the full deletion of *WAKs* in Arabidopsis. In contrast to previous results, flg22-induced ROS production in leaves of 3- to 4-wk-old Arabidopsis plants were not affected in *wakΔ2* in comparison with Col-0 under our conditions (Fig. 5, A and B). As expected, flg22-induced ROS production was dependent on the receptor FLAGELLIN-SENSING 2 (FLS2) and its co-receptor BRASSINOSTEROID-INSENSITIVE 1-ASSOCIATED KINASE 1 (BAK1). In line with this, flg22-induced MAPK activation, ethylene production, and induced resistance against *P. syringae* were not reduced in the *wakΔ2* mutant in comparison with Col-0 (Fig. 5, C to E). These results indicate that the deletion of *WAKs* does not affect flg22-induced responses under our growth conditions.

The *wakΔ2* mutant had no obvious growth phenotype when grown on soil (Fig. 1G). The only *wak*-related growth phenotype previously observed was reduced root length when seedlings were grown on MS medium lacking sucrose, most pronounced on 1/6 MS (Kohorn et al. 2006, 2021). Therefore, to investigate whether elicitor-induced root growth inhibition is affected in the *wakΔ2* mutant, both Col-0 and *wakΔ2* plants were grown in the presence of 10 nm*At*pep1, 100 nm flg22 or without elicitor for 5 d. Elicitor-induced root growth inhibition was similar in Col-0 and *wakΔ2* for both flg22 and pep1 (Fig. 5F).

## Discussion

PTI is achieved by the recognition of diverse elicitor molecules as ligands for plasma membrane-resident pattern recognition receptors (PRRs) (DeFalco and Zipfel 2021). Cell walls are the first layer of defense against invading pathogens, many of which have evolved arsenals of enzymatic and mechanical means to degrade or penetrate the cell wall (Bacete et al. 2018; Dora et al. 2022). Thus, the integrity of the cell wall needs to be carefully monitored by sensor proteins. Several RKs have been proposed as PRRs that perceive cell wall breakdown products, including WAKs based on their ability to interact with pectin and its breakdown products (He et al. 1996; Decreux and Messiaen 2005; Decreux et al. 2006; Kohorn et al. 2009; Brutus et al. 2010). Yet, genetic evidence that WAKs function as bona fide OG receptor(s) was missing. Here, we have used the *wakΔ2* mutant, which lacks all 5 members of the WAK family, to demonstrate that none of the WAKs are required for responses to either short- or long-chain demethylated OGs in Arabidopsis.

Previously, the galacturonan-binding domain of WAKs was shown to bind both pectins and demethylesterified OG_9–15_ (Decreux and Messiaen 2005; Decreux et al. 2006). Additionally, chimeric WAK-EFR receptors were able to induce EFR-like responses upon OG treatment (Brutus et al. 2010). While our results indicate that WAKs are not genetically required for OG-induced responses, they do not contradict the ability of WAK ECDs to bind pectins or pectin breakdown products. Interestingly, the ECD of the malectin-like RK FERONIA was also recently reported to bind to pectin and pectin breakdown products (Feng et al. 2018; Lin et al. 2022; Tang et al. 2022), suggesting that this biochemical property might be true for several cell wall-anchored RKs without necessarily functioning as the true receptors for these carbohydrates.

OG_10–15_ were suggested to be produced during pathogen infection and to subsequently induce immune signaling (Ferrari et al. 2013; Xiao et al. 2024). Although demethylesterified OG_10–15_ are active as elicitors, recent evidence challenges their production *in planta* as most OGs produced during infection with *B. cinerea* or *Fusarium oxysporum* were acetyl- and methylesterified (Voxeur et al. 2019; Gámez-Arjona et al. 2022). While pectic fractions of various sizes and modifications show elicitor activity, the complexity of those *in planta*-produced OGs as well as the profile of crude extracts produced in the lab complicates the attribution of individual OG species to the elicitor activity (Liu et al. 2023). Regardless of the exact nature of *in planta* OG species, WAKs have been previously proposed as the receptors for demethylesterified OG_10–15_ based on in vitro binding studies and chimeric approaches, and we are here unable to confirm any corresponding genetic requirement for WAKs in OG_10–15_-induced signaling. Interestingly, electrostatic analysis of the WAK1 ECD predicted by AlphaFold revealed a negatively charged galacturonan-binding domain at apoplastic pH, contradicting the suggested binding of polyanionic de-esterified pectins (Lee and Santiago 2023).

While our data demonstrate that members of the WAK family are not required for OG-induced responses, it is possible that quantitative phenotype(s) are masked by persistent functional redundancy. WAKs are characterized by a galacturonan-binding domain and many WAKs contain one or more copies of EGF-like domains in their ECD (Verica and He 2002; Stephens et al. 2022). In addition to 5 WAKs, there are at least 21 WAK-likes (WAKLs) in Arabidopsis. To date, clear evidence is missing that WAKLs are also able to bind cell wall fragments (Kohorn 2016), with the exception of WAKL22/RESISTANCE TO FUSARIUM OXYSPORUM 1 and WAKL14 (Huerta et al. 2023; Ma et al. 2024). However, based on their phylogenetic relationship, WAKLs are obvious candidates to test for further genetic redundancy. Aside from a role in OG perception, WAKs were recently suggested to be involved in the regulation of other RK complexes during immunity, indicating that they might serve as accessory RKs of PRR complexes. In tomato and cotton, WAKs interact with and positively regulate PRR complexes and are required for full responsiveness to the corresponding elicitors (Wang et al. 2020; Zhang et al. 2020). In Arabidopsis, the *wakΔ* mutant was less sensitive to multiple elicitors in terms of ROS production (Kohorn et al. 2021). In contrast to these previous results, no quantitative reduction in OG- or flg22-induced ROS production could be observed in the *wakΔ2* mutant. Although this difference is striking, it might further underline the role of WAKs as accessory RKs under certain growth conditions rather than OG-perceiving receptors. While WAKs appear to interact with multiple elicitor-perceiving RKs, the exact mechanisms by which WAKs regulate immunity seem to differ between plant species or different WAKs.

WAKs are found across land plants, with the *WAK/WAKL* family expanded in monocots (de Oliveira et al. 2014; Kanyuka and Rudd 2019; Stephens et al. 2022; Zhang et al. 2023a; Ngou et al. 2024). Several *WAKs* or *WAKLs* have been identified as resistance genes and are required for basal resistance to pathogens in a variety of different crop plants (Diener and Ausubel 2005; Hurni et al. 2015; Zuo et al. 2015; Hu et al. 2017; Saintenac et al. 2018; Bot et al. 2019; Larkan et al. 2020; Li et al. 2020; Stephens et al. 2022; Zhang et al. 2023b; Dai et al. 2024; Zhong et al. 2024). While diverse roles and mechanisms for WAKs in plant immunity have been proposed, a possibility is that WAKs perceive pathogen-derived molecules. Indeed, Arabidopsis WAK3 was recently shown to be required for immune responses induced by bacterial harpins (Held et al. 2024), indicating that WAKs might indeed perceive microbial molecules. Additionally, 3 WAKs have also been demonstrated to exhibit a gene-for-gene interaction with specific pathogenic effectors in crops (Stephens et al. 2022). The WAK proteins Stb6 and Rlm9 provide resistance against *Zymoseptoria tritici* isolates expressing AvrSbt6 in wheat (*Triticum aestivum*) and *Leptosphaeria maculans* expressing AvrLm5-9 in oilseed rape (*Brassica napus* L.), respectively (Brading et al. 2002; Larkan et al. 2016, 2020). While no direct interaction could be detected between these fungal effectors and corresponding WAK resistance proteins (Saintenac et al. 2018; Larkan et al. 2020), intriguingly, a direct interaction has been observed between the maize (*Zea mays*) WAK protein Snn1 and the *Phaeosphaeria nodorum* effector protein *Sn*Tox1. Unlike most other WAKs studied thus far, Snn1 serves as susceptibility factor for *P. nodorum*, leading to disease in Snn1-expressing plants (Liu et al. 2012; Stephens et al. 2022; Shi et al. 2023). Maize qRgls1**/**WAKL^Y^ was also recently shown to confer quantitative disease resistance against gray leaf spot caused by the fungi *Cercospora zeae-maydis* and *C. zeina* (Zhong et al. 2024). Notably, an aqueous extract of *C. zeina* hyphae and spores was sufficient to induce WAKL^Y^-dependent ROS production suggesting that WAKL^Y^ perceives a fungal ligand.

Altogether, there is emerging evidence that WAKs may perceive diverse molecules of microbial origin and orchestrate both broad-spectrum and race-specific resistance (Kanyuka and Rudd 2019), which is consistent with our evidence that they do not function as bona fide OG receptor(s) in Arabidopsis. However, the mechanisms by which WAKs contribute to immunity and their true ligand(s) remain to be characterized.

## Materials and methods

### Plant growth

Arabidopsis seeds were surface-sterilized using ethanol, plated on 0.5 MS medium (1% sucrose, pH 5.8, 0.9% phytoagar), stratified for 48 h in the dark at 4 °C, and grown at 22 °C under a 16-h photoperiod (120 μmol × s^−1^ × m^−2^ illumination). For assays in adult plants, including ROS production, pathogen infection, and callose deposition, seedlings were transferred to soil after 7 to 10 d growth on plates. Plants were grown in short-day cycles (10-h light/14-h dark, 60% humidity, 20 °C) for an additional 2 to 3 wk. For assays with seedlings, including MAPK activation, RNA extraction, seedling growth inhibition, and root growth inhibition, these were transferred 5 d after exposure to light to liquid MS and grown there for 10 d. Mutants were generated in the *A. thaliana* Columbia (Col-0) ecotype, and primers for genotyping are given in Supplementary Table S1.

### CRISPR–Cas9 mutagenesis

The *WAK4* and *WAK2* oligonucleotides used as templates for SgRNA-targeted sites (GCTGT TTCGTTATTGTTAAATGG) 432 bp 5′ to the *WAK4* ATG start codon and (GGGGAGATTGAACAC TTGCTCGG) 77 bp 5′ to the *WAK2* stop codon were each cloned into pSkAtu26 (Feng et al. 2013). These 2 expression Sg cassettes were then cloned into pCambia1302 that also had a pOLE1-RFP cassette inserted into the ASN718 site by PCR cloning (Shimada et al. 2010).

The T1, RFP^+^ (expressing linked CAS9 and sgRNAs) were screened for a deletion by PCR using primers flanking the deletion, and then, T2 RFP^−^ plants were screened again by PCR to isolate a plant with a deletion but not expressing CAS9 or the sgRNAs. These isolates were self-crossed to generate a homozygous deletion.

### RNA extraction and real-time quantitative PCR analysis

Total RNA was extracted from 2-wk-old liquid-grown seedlings. For quantitative PCR analysis, total RNA was extracted using TRI reagent (Sigma-Aldrich). To remove genomic DNA, samples were treated with TURBO DNA-free Kit (Thermo Fisher Scientific). cDNA synthesis was performed using 1 µg of DNA-free RNA sample with RevertAid First Strand cDNA Synthesis Kit (Thermo Fisher Scientific) according to the manufacturer’s protocol. RT-qPCR analysis was performed using diluted cDNA as a template for PowerUp SYBR Green (Applied Biosystems) with the primers provided in Supplementary Table S1. Transcripts were normalized to the house-keeping gene *UBOX*. Undetermined CT values in the *wakΔ2* were set to 40. 2^−ΔCT^ values were calculated. For each independent repetition (*n* = 2), 3 biological replicates with each 2 seedlings were harvested for each genotype.

For RNA sequencing, total RNA of 2-wk-old seedlings harvested was extracted using the FavorPrep Plant Total RNA Purification Mini Kit (FAVORGEN) according to the manufacturer’s manual. DNA digest was performed on column using TURBO DNase (Thermo Fisher) according to the manufacturer’s manual. mRNA library preparation (polyA enrichment) and sequencing were performed with Novogene (Munich, Germany, PE150, 6G raw data). Raw reads have been deposited in the European Nucleotide Archive under the accession number PRJEB80751. For each genotype analyzed, 4 seedlings were pooled for each independent repetition (*n* = 4).

### MAPK activation

MAPK activation was performed as previously described (Mühlenbeck et al. 2023). Five-day-old seedlings were transferred into 24-well plates containing 1 mL of liquid 0.5 mS (1% sucrose). Two seedlings per well were grown there for another 10 to 12 d. Seedlings were treated with water (mock), 100 µg/mL OG_10–15_ (Elicityl, GAT114), 100 µg/mL OG_3_ (GalA3, Megazyme), or 1 µM flg22 and harvested at each time point as indicated in figure captions. Total proteins were extracted from 2 pooled seedlings using extraction buffer (50 mm Tris pH 7.5 (HCl), 150 mm NaCl, 10% (v/v) glycerol, 2 mm EDTA, 1 mm homemade PPI (equivalent to Sigma-Aldrich Protease Inhibitor Cocktail P9599), 1 mm NaF, 1 mm sodium orthovanadate, 2 mm sodium molybdate, 4 mm sodium tartrate, 1% (v/v) IGEPAL CA630, 5 mm DTT). Proteins were analyzed by SDS–PAGE and immunoblotting using p44/42 MAPK antibody (Cell Signaling Technology, Catalog # 9101L). Three independent repetitions were performed for this experiment.

### Seedling growth inhibition

Seedling growth inhibition assays were performed as previously described (Abarca et al. 2021). Briefly, 5-d-old Arabidopsis seedlings were transferred to 48-well plate with 1 seedling per well. Each well contained either 500 µL 0.5 liquid MS with or without 200 µg/mL OG_10–15_ (Elicityl, GAT114). After 10 d of growth in the presence of the respective elicitor, individual seedling weight was assessed using an analytical balance. For each genotype and treatment combination, 12 seedlings were measured per genotype and treatment in each independent experiment (*n* = 3).

### Root growth inhibition

Five-day-old seedlings were transferred from solid MS plates to 12-well plate with 6 seedling per well. Each well contained 4 mL of liquid MS supplemented with mock (sterile ddH_2_O), 10 nm*At*pep1, or 100 nm flg22. After 5 d of treatment, seedlings were transferred to MS plates and imaged (*n* = 6 per genotype and treatment). The experiment was performed twice. Root lengths were quantified with ImageJ.

### Ethylene production

Four- to six-wk-old Arabidopsis leaves were cut into 3-mm slices and floated on water overnight. For each sample, 3 leaf slices were transferred to a 6-mL glass tube containing 200 µL MES buffer (pH 5.7), followed by adding either water control or the elicitor to a final concentration of 1 µM. Vials were closed with a rubber septum, and ethylene production in the free air space was measured by gas chromatography (Shimadzu, GC-14A) after 3 h of incubation. Three independent repetitions were performed.

### ROS production

Twelve to sixteen leaf discs of 6 to 8 3- to 4-wk-old plants were taken (4 mm Ø) per genotype and treatment. These leaf discs were placed with the abaxial side down into a well of a white polystyrene 96-well plate containing 100 μL ddH_2_O and recovered overnight. The next day, the water was replaced by a solution containing 20 µg/mL horseradish peroxidase (HRP, Sigma), luminol (17 µg/mL), and elicitor (100 nm for flg22, 100 µg/mL OG_10–15_ (Elicityl, GAT114), as stated). Luminescence was immediately measured for 60 min using a charge-coupled device camera (Photek Ltd, East Sussex, UK). This experiment was repeated 3 times with different batches of plants and performed at different days.

### Callose deposition

Callose deposition assays were performed as described previously (Mason et al. 2020). Briefly, 4 leaves of 4- to 5-wk-old plants were syringe-infiltrated with either mock (ddH_2_O) or 100 µg/mL OG_10–15_ (Elicityl, GAT114). Twenty-four hours after infiltration, 16 to 32 leaf discs from 4 different plants were taken per genotype and treatment and collected in 24-well plates filled with 1 mL 100% EtOH until completely destained. Leaf discs were equilibrated in 1 mL 67 mm K_2_HPO_4_ (pH 12) for 60 min. Afterward, the tissue was stained using aniline blue (Acros Organics) staining solution 0.01% (w/v) aniline blue in 67 mm K_2_HPO_4_ (pH 12) for 60 min and washed with 67 mm K_2_HPO_4_ (pH = 12) for 60 min. Stained tissue was mounted in mounting solution (80% glycerol, 67 mm K_2_HPO_4_, pH 12) on microscope slides. Callose deposits were imaged using a Leica DM6000B and quantified with ImageJ. The experiment was repeated twice with different batches of plants and on different days.

### PR1 protein abundance

PR1 accumulation was assayed as previously described (Bender et al. 2021). Briefly, 3 leaves of 3-wk-old plants were infiltrated with mock (sterile ddH_2_O), 1 µM flg22, 100 µg/Ml, or OG_10–15_ (Elicityl, GAT114). Twenty-four hours after infiltration, leaves were harvested in 1.5-mL tubes, snap-frozen in liquid nitrogen, and pulverized. Extraction buffer (50 mm Tris pH7.5 (HCl), 150 mm NaCl, 10% (v/v) glycerol, 2 mm EDTA, 1× plant protease inhibitor cocktail) was added, and protein concentration was adjusted by the Bradford assay. Normalized protein extracts were analyzed by SDS–PAGE (15%) and immunoblotting using PR1 antibodies (Agrisera, catalog # AS10 687). The experiment was repeated 3 times with different batches of plants and on different days.

### Induced resistance against *P. syringae*

Two leaves of 4- to 5-wk-old plants were infiltrated with 1 µM flg22 or 100 µg/mL OG_10–15_ (Elicityl, GAT114) or mock (sterile ddH_2_O). Freshly restreaked *P. syringae* pv tomato DC3000 was grown in liquid Kings B overnight and refreshed in a subculture the next morning for additional 1 to 2 h. Bacteria were infiltrated into pretreated leaves with an OD_600_ of 0.0002. Plants were covered for 2 d, after which 1 leaf disc was harvested per treated leaf (8 mm Ø) and pooled per plant. Leaf discs were ground in 10 mm MgCl_2_, thoroughly mixed, and diluted in a 1:10 series until 1:10^−6^. Samples were plated on LB plates. After 2 d of growth at 28 °C, colony forming units (CFU) were counted. Statistics were performed on log_10_ (CFU/cm^2^). For each independent experiment (*n* = 3), 6 to 8 plants were used per genotype and treatment.

### Induced resistance against *B. cinerea*

Four leaves of 4- to 5-wk-old plants were infiltrated with 100 µg/mL OG_10–15_ (Elicityl, GAT114) or mock (sterile ddH_2_O) in the morning. The next day, spores of *B. cinerea* BMM were collected in sterile ddH_2_O and the spores were counted using a counting chamber. At least 1 h prior infection, infection solutions were prepared with a final concentration 5 × 10^5^ spores/mL in 0.5 potato dextrose broth and incubated at RT. Five microliters of the *Botrytis* infection solution was dropped on the adaxial site next to the middle vein. Plant solid trays were filled with water, covered with a lid, and sealed with parafilm to produce high humidity. After 2 d after infection at dimmed light, leaves were detached, images were taken, and lesion size was measured using ImageJ. For each independent experiment (*n* = 4), 3 to 4 leaves from 6 plants were infected per genotype and treatment.

### Statistical analysis

The statistical analysis for data that were not normally distributed or where the variance between groups was not similar was performed applying the nonparametric Kruskal–Wallis multiple comparison test with Dunn’s post hoc test. For data that follow a normal distribution and similar variance between groups, 2-way ANOVA with Tukey’s post hoc test was applied. Statistical analysis was performed using the software R studio, and statistical data are provided in Supplementary Data Set 1.

### Accession numbers

Sequence data from this article can be found in the EMBL data libraries under the accession numbers listed in Supplementary Table S2.

## Supplementary Material

koae317_Supplementary_Data

## Data Availability

The data underlying this article are available in the article and in its online supplementary material. The RNAseq data are available in the EMBL data libraries under the accession number PRJEB80751.

## Supplementary data

The following materials are available in the online version of this article.

**Supplementary Table S1.** Primers used in this study.

**Supplementary Table S2.** RNA-seq accession numbers.

**Supplementary Table S3.** Accession numbers of the genes and proteins used in this study.

**Supplementary Data Set 1.** Statistical data.

## Dive Curated Terms

The following phenotypic, genotypic, and functional terms are of significance to the work described in this paper:

WAK1 Gramene: AT1G21250
WAK1 Araport: AT1G21250

WAK4 Gramene: AT1G21210
WAK4 Araport: AT1G21210

FLS2 Gramene: AT5G46330
FLS2 Araport: AT5G46330

WAK2 Gramene: AT1G21270
WAK2 Araport: AT1G21270

BAK1 Gramene: AT4G33430
BAK1 Araport: AT4G33430

PR1 Gramene: At2g14610
PR1 Araport: At2g14610

EDTA Gramene: Ethylene Diamine Tetraacetic Acid
EDTA Araport: Ethylene Diamine Tetraacetic Acid

AtPep1 Gramene: AT5G64900
AtPep1 Araport: AT5G64900

GhWAK7A Gramene: Gh_A02G0727
GhWAK7A Araport: Gh_A02G0727
