# Supplementary material for: Arabidopsis WALL-ASSOCIATED KINASES are not required for oligogalacturonide-induced signaling and immunity
Source: Plant Cell. 2024 Dec 12;37(1):koae317. doi: 10.1093/plcell/koae317 (PMC11684076; doi:10.1093/plcell/koae317)
Supplement: koae317_Supplementary_Data [file koae317_supplementary_data.zip › Supplementary_data_table13_suppDataSet.pdf]

Supplementary data

SUPPLEMENTARY TABLE 1 – Primers used in this study.

Target	Primer forward	Primer reverse
P1 – pair (*WAK4*)	ACTATCTCTTTGAGCGGCTCC	TTGGTATCAGCCTTGAAGCAC
P2 – pair (big deletion)	ACTATCTCTTTGAGCGGCTCC	ACTATCTCTTTGAGCGGCTCC
P3 -pair (*WAK3*)	CCAAGACGCCGATTATGATTAC	AGAAGATGAAGTTCCAGGAGGG
P4 - pair	CAAGCATGATAACCGAGAAGC	GATATGATGAAGAGCCCTGCG
WAK1-qPCR	ACCTTTCAGCTGGTTGCCAAGAC	TGGTGGTATCTAAGCGGTAACCAG
WAK2-qPCR	TGCCCATCTGGTTACCGCAAAG	AGAAGCCGATGGTGGTTCCAAG
WAK3-qPCR	ACCGTTCAGAGGGTTGCAAAGAC	ACTTACAATCGAAGCCTCCATCCC
WAK4-qPCR	CTTTGCCTCAGCCACGAAAGAG	TCTCGTTCATCACTTGGCCATC
WAK5-qPCR	TCGGACGGTTGCCAAGACATC	GGGCACTGACAATGGAAGCTTCC
UBOX	TGCGCTGCCAGATAATACACTATT	TGCTGCCCAACATCAGGTT

SUPPLEMENTARY TABLE 2 – RNAseq accession numbers

Study_accession_number	Run_accession_number	Sample_accession_number	description
PRJEB80751	ERR13804691	ERS21114706	Col-0_wt_1
PRJEB80751	ERR13804692	ERS21114707	Col-0_wt_2
PRJEB80751	ERR13804693	ERS21114708	Col-0_wt_3
PRJEB80751	ERR13804694	ERS21114709	Col-0_wt_4
PRJEB80751	ERR13804695	ERS21114710	Col-0_wakd2_1
PRJEB80751	ERR13804696	ERS21114711	Col-0_wakd2_2
PRJEB80751	ERR13804697	ERS21114712	Col-0_wakd2_3
PRJEB80751	ERR13804698	ERS21114713	Col-0_wakd2_4

SUPPLEMENTARY TABLE 3 – Accession numbers of the genes and proteins used in this study.

Gene name / Protein name	Accession number

WAK1	AT1G21250
WAK2	AT1G21270
WAK3	AT1G21240
WAK4	AT1G21210
WAK5	AT1G21230
WAKL1	AT1G16120
WAKL2	AT1G16130
WAKL3	AT1G16140
WAKL4	AT1G16150
WAKL5	AT1G16160
WAKL6	AT1G16110
WAKL7	AT1G16090
WAKL8	AT1G16260
WAKL9	AT1G69730
WAKL10	AT1G79680
WAKL11	AT1G19390
WAKL12	AT1G22720
WAKL13	AT1G17910
WAKL14	AT2G23450
WAKL15	AT3G53840
WAKL16	AT3G25490
WAKL17	AT4G31100
WAKL18	AT4G31110
WAKL20	AT5G02070
WAKL21	AT5G66790
WAKL22	AT1G79670

SUPPLEMENTARY DATA SET 1. - Statistical data

SHEET 1 – Figure 2

Figure 2 B - ROS

Kruskal-Wallis Test		
**chi-squared**	**df**	**p-value**
0.94829	1	0.3302

Figure 2 D - Ethylene

**Kruskal-Wallis Test**		
chi-squared	df	p-value
20.99	3	0.0001058

**Dunn's multiple comparisontest**			
Comparison	Z	P.unadj	p.adj
Col-0_Mock - Col-0_OGs	-2.86578853	0.0041597201	0.024958321
Col-0_Mock - wakC2_Mock	0.04276995	0.9658849216	1.0
Col-0_OGs - wakC2_Mock	2.91264066	0.0035838675	0.021503205
Col-0_Mock - wakC2_OGs	-3.49048358	0.0004821472	0.002892883
Col-0_OGs - wakC2_OGs	-0.69843030	0.4849081355	1.0
wakC2_Mock - wakC2_OGs	-3.53733571	0.0004041856	0.002425113

SHEET 2 – Figure 3

Figure 3 B - Callose

**Kruskal-Wallis Test**		
chi-squared	df	p-value
93.593	3	< 2.2e-16

**Dunn's multiple comparisontest**			
Comparison	Z	P.unadj	p.adj
Col-0_Mock - Col-0_OGs	-6.92000645	4,52E-12	2,71E-11
Col-0_Mock - wakC2_Mock	-0.11002500	9,12E-01	1,00E+00
Col-0_OGs - wakC2_Mock	6.65117594	2,91E-11	1,74E-10
Col-0_Mock - wakC2_OGs	-7.02457574	2,15E-12	1,29E-11
Col-0_OGs - wakC2_OGs	-0.02910355	9,77E-01	1,00E+00
wakC2_Mock - wakC2_OGs	-6.74884079	1,49E-11	8,94E-11

Figure 3 C - SGI

**Kruskal-Wallis Test**		
chi-squared	df	p-value
24,9710	3	1,57E-05

**Dunn's multiple comparisontest**			
Comparison	Z	P.unadj	p.adj
Col-0_Mock - Col-0_OGs	2.6490399	8,07E-03	0.0161441612
Col-0_Mock - wakC2_Mock	-1.5964806	1,10E-01	0.1324578341
Col-0_OGs - wakC2_Mock	-4.2641105	2,01E-05	0.0001204201

Col-0_Mock - wakC2_OGs	2.4026472	1,63E-02	0.0244153203
Col-0_OGs - wakC2_OGs	-0.2649826	7,91E-01	0.7910228545
wakC2_Mock - wakC2_OGs	4.0275918	5,64E-05	0.0001690532

SHEET 3 - Figure 4

Figure 4 A

Kruskal-Wallis Test		
chi-squared	df	p-value
63.912	3	8,57E-14

Dunn's multiple comparisontest			
Comparison	Z	P.unadj	p.adj
Col-0_Mock - Col-0_OGs	5.2816438	1,28E-07	7,68E-07
Col-0_Mock - wakC2_Mock	-0.6284563	5,30E-01	1,00E+00
Col-0_OGs - wakC2_Mock	-5.8807333	4,08E-09	2,45E-08
Col-0_Mock - wakC2_OGs	5.3969215	6,78E-08	4,07E-07
Col-0_OGs - wakC2_OGs	0.1476919	8,83E-01	1,00E+00
wakC2_Mock - wakC2_OGs	5.9919600	2,07E-09	1,24E-08

Figure 4 C

Two-way ANOVA					
	Df	Sum Sq	Mean Sq	F value	Pr(>F)
genotype	1	1.15	1.153	2.481	0.119
treatment	1	15.16	15.162	32.630	0,000000206
genotype:treatment	1	0.04	0.043	0.093	0.762
Residuals	76	35.51	0.465		

Tukey Post-hoc test				
Comparison	diff	lwr	upr	p adj
Col-0_Mock - wakC2_Mock	0.1936746	-0.3725545	0.75990374	0.8056349
Col-0_Mock - Col-0_OGs	-0.9170829	-1.4833120	-0.35085376	0.0003390
Col-0_Mock - wakC2_OGs	-0.6306065	-1.1968356	-0.06437743	0.0230006
Col-0_OGs - wakC2_Mock	-1.1107575	-1.6769866	-0.54452840	0.0000116
wakC2_Mock - wakC2_OGs	-0.8242812	-1.3905103	-0.25805207	0.0014963
Col-0_OGs - wakC2_OGs	0.2864763	-0.2797528	0.85270543	0.5475536

SHEET 4 - Figure 5

Figure 5 B

Kruskal-Wallis Test		
chi-squared	df	p-value
49	3	1,23E-10

Dunn's multiple comparisontest			
Comparison	Z	P.unadj	p.adj
Col-0 - bak1-5/bkk1-1	-4.4374532	9,10E-06	5,46E-05
bak1-5/bkk1-1 - fls2/efr/cerk1	0.5361186	5,92E-01	1,00E+00
Col-0 - fls2/efr/cerk1	5.0646022	4,09E-07	2,46E-06
bak1-5/bkk1-1 - wakΔ2	-4.7967747	1,61E-06	9,67E-06
Col-0 - wakΔ2	-0.4521362	6,51E-01	1,00E+00
fls2/efr/cerk1 - wakΔ2	-5.4239236	5,83E-08	3,50E-07

Figure 5 D

Kruskal-Wallis Test		
chi-squared	df	p-value
53	7	3,91E-09

Dunn's multiple comparisontest			
Comparison	Z	P.unadj	p.adj
bak1-5/bkk1_flg22 - bak1-5/bkk1_Mock	1.35921637	1,74E-01	1,00E+00
bak1-5/bkk1_flg22 - Col-0_flg22	-3.70689215	2,10E-04	5,87E-03
bak1-5/bkk1_Mock - Col-0_flg22	-4.67476150	2,94E-06	8,24E-05
bak1-5/bkk1_flg22 - Col-0_Mock	-0.18463344	8,54E-01	1,00E+00
bak1-5/bkk1_Mock - Col-0_Mock	-1.40933561	1,59E-01	1,00E+00
Col-0_flg22 - Col-0_Mock	3.13091169	1,74E-03	4,88E-02
bak1-5/bkk1_flg22 - fls2/efr_flg22	0.20203441	8,40E-01	1,00E+00
bak1-5/bkk1_Mock - fls2/efr_flg22	-1.17851130	2,39E-01	1,00E+00
Col-0_flg22 - fls2/efr_flg22	3.90892655	9,27E-05	2,60E-03
Col-0_Mock - fls2/efr_flg22	0.36533850	7,15E-01	1,00E+00
bak1-5/bkk1_flg22 - fls2/efr_Mock	0.59318402	5,53E-01	1,00E+00
bak1-5/bkk1_Mock - fls2/efr_Mock	-0.69928866	4,84E-01	1,00E+00
Col-0_flg22 - fls2/efr_Mock	3.90872915	9,28E-05	2,60E-03
Col-0_Mock - fls2/efr_Mock	0.71004695	4,78E-01	1,00E+00
fls2/efr_flg22 - fls2/efr_Mock	0.41247896	6,80E-01	1,00E+00
bak1-5/bkk1_flg22 - wakC2_flg22	-4.11096096	3,94E-05	1,10E-03
bak1-5/bkk1_Mock - wakC2_flg22	-5.03617163	4,75E-07	1,33E-05
Col-0_flg22 - wakC2_flg22	-0.40406881	6,86E-01	1,00E+00

Col-0_Mock - wakC2_flg22	-3.49232182	4,79E-04	1,34E-02
fls2/efr_flg22 - wakC2_flg22	-4.31299536	1,61E-05	4,51E-04
fls2/efr_Mock - wakC2_flg22	-4.27013928	1,95E-05	5,47E-04
bak1-5/bkk1_flg22 - wakC2_Mock	-0.11392276	9,09E-01	1,00E+00
bak1-5/bkk1_Mock - wakC2_Mock	-1.34478588	1,79E-01	1,00E+00
Col-0_flg22 - wakC2_Mock	3.20162237	1,37E-03	3,83E-02
Col-0_Mock - wakC2_Mock	0.06454972	9,49E-01	1,00E+00
fls2/efr_flg22 - wakC2_Mock	-0.29462783	7,68E-01	1,00E+00
fls2/efr_Mock - wakC2_Mock	-0.64549722	5,19E-01	1,00E+00
wakC2_flg22 - wakC2_Mock	3.56303250	3,67E-04	1,03E-02

Figure 5 E

Kruskal-Wallis Test		
chi-squared	df	p-value
95	5	< 2.2E-16

Dunn's multiple comparisontest			
Comparison	Z	P.unadj	p.adj
bbc_flg22 - bbc_mock	-0.72653789	4,68E-01	5,39E-01
bbc_flg22 - Col-0_flg22	6.56634401	5,16E-11	2,58E-10
bbc_mock - Col-0_flg22	7.29288189	3,03E-13	4,55E-12
bbc_flg22 - Col-0_mock	3.08262921	2,05E-03	2,80E-03
bbc_mock - Col-0_mock	3.80916710	1,39E-04	2,99E-04
Col-0_flg22 - Col-0_mock	-3.48371480	4,95E-04	9,27E-04
bbc_flg22 - wakC2_flg22	6.40207421	1,53E-10	5,75E-10
bbc_mock - wakC2_flg22	7.11923702	1,09E-12	8,14E-12
Col-0_flg22 - wakC2_flg22	-0.07953923	9,37E-01	9,37E-01
Col-0_mock - wakC2_flg22	3.35922253	7,82E-04	1,30E-03
bbc_flg22 - wakC2_mock	2.54861240	1,08E-02	1,35E-02
bbc_mock - wakC2_mock	3.27515029	1,06E-03	1,58E-03
Col-0_flg22 - wakC2_mock	-4.01773160	5,88E-05	1,76E-04
Col-0_mock - wakC2_mock	-0.53401681	5,93E-01	6,36E-01
wakC2_flg22 - wakC2_mock	-3.88634851	1,02E-04	2,54E-04

Figure 5 F

Two-way ANOVA					
	Df	Sum Sq	Mean Sq	F value	Pr(>F)
genotype	1	0.00	0.001	0.006	0.939
treatment	2	47.74	24	192	<2E-16
genotype:treatment	2	0.01	0.005	0.037	0.963
Residuals	66	8.20	0.124		

Tukey Post-hoc test				
Comparison	diff	lwr	upr	p adj
wakC2:flg22-Col-0:flg22	-0.006461538	-0.4122740	0.3993509	1
Col-0:mock-Col-0:flg22	1.069224359	0.6550437	1.4834050	0.0000000
wakC2:mock-Col-0:flg22	1.035580420	0.6117231	1.4594377	0.0000000

Col-0:pep1-Col-0:flg22	-0.995025641	-1.4092063	-0.5808450	0.0000000
wakC2:pep1-Col-0:flg22	-0.971783217	-1.3956405	-0.5479259	0.0000001
Col-0:mock-wakC2:flg22	1.075685897	0.6615053	1.4898665	0.0000000
wakC2:mock-wakC2:flg22	1.042041958	0.6181847	1.4658993	0.0000000
Col-0:pep1-wakC2:flg22	-0.988564103	-1.4027447	-0.5743835	0.0000000
wakC2:pep1-wakC2:flg22	-0.965321678	-1.3891790	-0.5414644	0.0000001
wakC2:mock-Col-0:mock	-0.033643939	-0.4655199	0.3982320	0.9999103
Col-0:pep1-Col-0:mock	-2.064250000	-2.4866330	-1.6418670	0.0000000
wakC2:pep1-Col-0:mock	-2.041007576	-2.4728835	-1.6091316	0.0000000
Col-0:pep1-wakC2:mock	-2.030606061	-2.4624820	-1.5987301	0.0000000
wakC2:pep1-wakC2:mock	-2.007363636	-2.4485283	-1.5661990	0.0000000
wakC2:pep1-Col-0:pep1	0.023242424	-0.4086335	0.4551184	0.9999857
