# Supplementary material for: Arabidopsis WALL-ASSOCIATED KINASES are not required for oligogalacturonide-induced signaling and immunity
Source: Plant Cell. 2024 Dec 12;37(1):koae317. doi: 10.1093/plcell/koae317 (PMC11684076; doi:10.1093/plcell/koae317)
Supplement: koae317_Supplementary_Data [file tpc.00387.2024-s05.pdf › tpc.00387.2024-s05.pdf]

THE PLANT CELL

Arabidopsis WALL-ASSOCIATED KINASES are not required for oligogalacturonide-induced signaling and immunity

Laura Herold, Jana Ordon, Chenlei Hua, Bruce Kohorn, Thorsten Nurnberger, Thomas DeFalco, and Cyril Zipfel

Review Timeline:

Submission Date:	18-Apr-2024
Editorial Decision:	23-May-2024
Revision Received:	23-Aug-2024
Editorial Decision:	24-Sep-2024
Revision Received:	25-Oct-2024
Accepted:	31-Oct-2024

Prof. Dr. Cyril Zipfel
Universitat Zurich
Zurich, Select One
Switzerland

Dear Dr. Zipfel, dear Cyril,

We have received reviews of your manuscript entitled "Arabidopsis WALL-ASSOCIATED KINASES are not required for oligogalacturonide-induced signaling and immunity." Thank you for submitting your best work to The Plant Cell. The editorial board agrees that the work you describe is substantive, falls within the scope of the journal, and may become acceptable for publication, pending revision and potential re-review.

As you will see, all three referees are supportive of publication, but flag a number of issues with the presentation and data or controls, which we would ask you to address. If you consider specific suggestions to be unrealistic or otherwise unreasonable, please indicate this clearly in your point-by-point response to reviewers' comments.

More generally, please contact us if there are ambiguous comments or if you wish to discuss the revision.

Given the nature of the comments, we are offering you 60 days from when we have issued this decision to complete the revision. If a revision is not returned within this time frame, and if you have not been granted an extension, we will withdraw the manuscript, which will leave you free to submit the work elsewhere. If you need an extension, we encourage you to contact us at any point before the 60 days have passed.

When you are ready to submit the revised version, please upload a highlighted copy that indicates all changes made in response to the editor and reviewer recommendations. Include an itemized list of all changes made in response to each of the reviewer's suggestions in the "Response to Reviewers" section; please note that reviewers do not have access to your cover letter, nor was this decision letter shared with them.

Thank you for the privilege of reviewing your work. We look forward to receiving your revised manuscript.

Sincerely,

The Plant Cell Editorial Board

(Please note that due to a potential conflict of interest, EIC Blake Meyers did not participate in evaluating this manuscript).

Please note the following:

-The Plant Cell now requires authors to complete and submit an author revisions checklist upon submission of a revised manuscript. The aim of the checklist is to aid authors in preparing a high-quality manuscript, facilitate the review and assessment of revised manuscripts, and help to ensure that journal standards are maintained across the board. If your manuscript is accepted, the completed checklist will be published as supplemental material attached to the article online. Please download a copy of the checklist (pdf fillable form) at this link, for submission with your revised manuscript: https://tpc.msubmit.net/html/Author_Revisions_Checklist.pdf.

-Supplemental materials should be restricted to large datasets and tables, presentation of replicates, and validation of reagents, methods, or genotypes. Any data that are used to support the major claims must be in the main manuscript. Supplemental figure legends must indicate what figure in the main manuscript is supported by the supplemental data presented. Please justify how each of the supplemental figures meet the criteria.

-Sampling methods and nature of "biological replicates" should be described precisely (i.e. different plants, parts of plants, pooled tissue, independent pools of tissue, sampled at different times, etc), along with a clear description of and rationale for any statistical analyses conducted. The reader should know exactly what was sampled; what forms the basis of the calculation of any means and statistical parameters reported. This is also necessary to ensure that proper statistical analysis was conducted.

-Want to add this revision deadline to your calendar? Click below!

-- Reviewer comments:

Reviewer #1 (Comments for the Author):

Herol et al. present a novel view on the role of Arabidopsis WALL-ASSOCIATED KINASES (WAKs) in OGs-induced defence responses. Their extensive research, using various hallmarks of early and late pattern-triggered immunity responses (PTIs), demonstrates that WAKs are not required for OG-induced signalling and immunity. Therefore, the authors firmly concluded that WAKs are not what they call bona fide OG receptors.

Several concerns and suggestions should be addressed before this paper is considered for publication in The Plant Cell. They are listed below in the order of the manuscript.

1. Abstract, line 27, The term "novel deletion mutant" is somewhat cryptic and does not clearly convey that this is the quintuple CRISPR cas deletion line. Clarity in this term is crucial for the readers' understanding of the research.

2. Results section, Generation of the wakΔ2 mutant. The manuscript does not clarify whether the absence of the entire WAK cluster was confirmed or tested with sequencing. Additionally, the genetic background used to generate wak2 is not specified. These details are important for understanding the reliability of the results and the potential for replication.

3. Results, line 113 and onwards- This sentence should be revised and rewritten.

4. Results, line 118 and onwards, I am slightly conflicted with this statement. The authors claim that if a particular protein is a receptor for a specific ligand, the loss of function mutant should not display any responses upon elicitation with that ligand. While this is primarily shown for ligands in the form of small peptides, where specific amino acids sequence is recognized and bound by the ligand-perceiving receptor, I would be more cautious with one-to-one translating this trend to all types of ligands (hormones, lipids, pectin-derived oligogalacturonides (OGs)). It could be very much that there are different biologically active species of ligand molecules that can have partial affinity towards either, as mentioned by the authors, bona fide receptors or some homologs.

5. Results, line 125 and onwards-Could this be driven by growing plants in two different photoperiods? The data presented in this manuscript were obtained in the short-day conditions of 10 hours of light and 14 hours of darkness, and the authors of Kohorn et al., 2021 used the long-day photoperiods of 16 hours of light and 8 hours of darkness.

6. I was wondering if the development of the wakΔ2 mutant line was undistinguishable from wild-type plants through the entire life cycle. In line with this, in Figure 3C, I would like to see the absolute fresh weight of the seedlings grown in the mock conditions.

7. Figure 2- What do the authors think about the primed MAPK activity in the wakΔ2 mutant line upon OG3 treatment at 0 minutes? Is 0 minutes corresponding to mock-only treatment or just immersing the seedlings in 100 µg/mL OG10-15 or 100 µg/mL OG3? If the latest one, mock treatment is missing.

8. Results, line 143 and onwards. While in plant pathology, callose deposition is typically viewed after hours, it is also an early response to MAMP challenge or pathogen infection that occurs within minutes. I suggest reviewing recent works by the plasmodesma community for more information and references.

9. What do the authors think about the enhanced expression of PR1 in wakΔ2 mutant upon OG10-15 compared to Col-0? The results section states, "Both flg22 and OG10-15 -induced PR1 accumulation was not affected in wakΔ2 (FIGURE 3D). OG3 induced very weak PR1 accumulation; however, no difference in PR1 accumulation could be detected between Col-0 and wakΔ2 (FIGURE 3D)." Is that the case that no difference in PR1 accumulation in the wak2 versus Col was detected?

10. As much as I am impressed by the clear and high-quality data that the authors presented in this manuscript falling into one coherent story demonstrating that the CRISPR cas quintuple does not display any altered defence response upon OGs or other elicitors induction in comparison to Columbia O plants... It makes me wonder how peculiar these observations are. Removing the entire WAK cluster makes (keeps?) plants indistinguishable from Col-0 when checking on different developmental stages and using various methods to monitor broadly understood PTI responses. I would like to ask the authors to consider the following
- demonstrating the sequencing results demonstrating that, indeed, the entire cluster of 5 WAK members is not present in the wakΔ2 mutant line
- the quintuple lines of (quintuple1 and quintuple2) were also described in in Lui et al., 2023. Would it be possible to validate some of the results obtained for wakΔ2 using the before-mentioned mutant lines to validate some of the obtained results?

11. The authors of this paper presented that in the wakΔ2 defence responses are not changed compared to the Col-0 control. This makes me immediately curious: What, then, is being changed? Does the absence of WAK cluster genes trigger any compensation mechanisms? Are the WAKL genes undergoing any transcriptional changes?

I was wondering if it would be possible to provide transcriptional data for wakΔ2 versus Col-0.

12. General comment 1: The authors use plants at different developmental stages for different experimental approaches. It would be valuable for the reader if this were stated more explicitly.

13. General comment 2: For most, if not for all, the figures, it seems completely senseless to present and annotate individual biological/technical repeats. It is not possible to distinguish them on the box plots anyway, and addicting this additional information to the figure makes it very difficult to read.

14. General comment 3: The arrangement of the figure panels seems disproportional for some figures, like Figure 2 and Figure 4.

Reviewer #2 (Comments for the Author):

This manuscript by Herold et al. presents a provocative finding that challenges the accepted role of Arabidopsis WALL-ASSOCIATED KINASES (WAKs) in perceiving oligogalacturonides (OGs) and initiating immune responses. The study uses genetic evidence to demonstrate that WAKs are not essential for OG-induced signalling and immunity in Arabidopsis, suggesting that WAKs have a different function and, potentially, that other receptors might be involved in this process. This finding challenges the well-accepted notion in the field that WAKs are OG receptors, and therefore their claims must be carefully presented.
One of the critical gaps in the presented data is the source and quality control of the OGs used in the experiments. The authors rely on a single OG commercial source without an explicit quality control, which might lead to misleading conclusions if the OG preparations are contaminated or functionally inconsistent. I suggest incorporating a quality check (e.g. GC/MS, HPLC/MS, HP-SEC-MS) to confirm that these preparations contain only OG. Additionally, employing a secondary source of OGs, whether commercial or derived from controlled digestion of homogalacturonan, would provide a robust foundation to validate the observed responses.
Another missing element in the experimental setup is the measurement of a PTI that I am sure the authors are familiar with: Ca2+ influx. This is a well-documented early signalling event in plant immune responses triggered by different MAMPs/DAMPs including OGs. I suggest including this in Figure 2, in order to provide a more comprehensive view of the signalling cascades involved.
The western blots presented in the manuscript to analyse early and late PTI display several inconsistencies and technical issues that could affect the interpretation of the results. Figure 2C includes BAK1 as a loading control, but not 2D (despite the legend indicating that it was used in both). For the OG 10-15 treatment in the wakΔ2 mutant, there is a band possibly for MAPK4-11, which is not usual for OGs. For OG3 in the wakΔ2 mutant it seems to be already some activation at t0. In Figure 3D, there is a surprising and interesting stronger activation in the wakΔ2 mutant in comparison to the wild type, however the text says that no difference could be detected (line 157 - 158).
The manuscript also raises questions regarding the comparative analysis of different mutants. The observation that a partial deletion mutant has a stronger phenotype (at least in terms of ROS production) than a complete delection is intriguing. I wonder why the authors did not include wakΔ (the partial deletion) as a control/comparison. Regarding ROS, in Figure 2A there seems to be a difference in the peaks of the two genotypes, although the effect is somehow buffered in the total RLU measurements (Figure 2B). Perhaps it is a difference that the authors should at least acknowledge. Furthermore, the absence of observable phenotypes in mutants with no WAK expression contradicts earlier studies using antisense RNA where such modifications resulted in lethality or clear phenotypic changes. This contradiction should be addressed, possibly suggesting alternative explanations or experimental limitations that could account for these differences.
Minor: some references are missing from the reference list, for example these relative to FERONIA (Feng et al 2018, Tang et al 2022, Lin et al 2022).
In any case, the manuscript is interesting and well-written, and the results could potentially open new avenues for research into the real receptors of OGs and the biological relevance of WAKs. However, these findings must be

supported by rigorous experimental validation to gain wider acceptance in the field.

Reviewer #3 (Comments for the Author):

The paper by Herold et al. is of major interest as it revolutionizes the current textbook model describing WAK receptors as GalA10-GalA15 receptors. While I have no comments on the experiments performed, I do have comments regarding the way the paper describes and names the OGs they tested.
The authors specify that oligogalacturonides are 'pectin-derived oligogalacturonides.' For their experiments, the 'authors' specifically use to a mixture of oligogalacturonides ranging from DP10 to DP15, produced from polygalacturonic acid, i.e., from chemically fully demethylesterified pectins. Since they discuss the fact that endogenous OGs detected in vivo display chemical decorations such as methylesterification and/or acetylation (l. 230-231) and that these methyl- and acetylated OGs could be WAK substrates, it should be clear both in the text and in the title to which OGs the authors are referring, i.e., deca to pentadeca fully demethylesterified OGs as it is done line 228. Therefore, I would recommend using GalA10-GalA15 instead of OG10-15 to avoid any confusion as it has been done on line 83 for GalA3.

Other comment :
Line 45. OGs are generated from demethylesterified pectins.
It's not accurate. When digested with a pectin lyase, methylesterified OG are produced from highly methylesterified pectins.

Reviewing Editor comments:

Dear Editor,

We would like to express our sincere appreciation for the reviewers' comments and constructive suggestions.
We have performed additional experiments and modified text sections to address the referees' concerns. We also provide a point-by-point response to the comments received below.
We therefore hope that our manuscript will now be acceptable for publication in The Plant Cell.

Yours sincerely,
Cyril Zipfel, on behalf of all authors

Response to Reviewer #1

Herold et al. present a novel view on the role of Arabidopsis WALL-ASSOCIATED KINASES (WAKs) in OGs-induced defence responses. Their extensive research, using various hallmarks of early and late pattern-triggered immunity responses (PTIs), demonstrates that WAKs are not required for OG-induced signalling and immunity. Therefore, the authors firmly concluded that WAKs are not what they call bona fide OG receptors.

Several concerns and suggestions should be addressed before this paper is considered for publication in The Plant Cell. They are listed below in the order of the manuscript.

1. Abstract, line 27, The term "novel deletion mutant" is somewhat cryptic and does not clearly convey that this is the quintuple CRISPR cas deletion line. Clarity in this term is crucial for the readers' understanding of the research.
>> We thank the reviewer for this suggestion and changed the text in the revised manuscript to "using a novel CRISPR mutant in which all five *WAK* genes were deleted" (line 27).

2. Results section, Generation of the wakΔ2 mutant. The manuscript does not clarify whether the absence of the entire WAK cluster was confirmed or tested with sequencing. Additionally, the genetic background used to generate wak2 is not specified. These details are important for understanding the reliability of the results and the potential for replication.
>> We thank the reviewer for this comment. The *wakΔ2* mutant was generated in the *A. thaliana* ecotype Col-0 background as mentioned in "Material and Methods" in the section "Plant growth". We have adapted the text in the revised manuscript accordingly to highlight in the Results section that the mutant was generated in the Col-0 ecotype (lines 103-104).
To confirm the deletion, we sequenced the borders of the gene cluster. We have now added the sequencing chromatogram to Figure 1 and edited the text accordingly (lines 105-106). Whole genome sequencing was performed for the *wakΔ* mutant (Kohorn *et al*., 2021; https://doi.org/10.1371/journal.pone.0251922) that was used for the deletion of the 5' end of *WAK4* already showing the deletion of most of the cluster.

3. Results, line 113 and onwards - This sentence should be revised and rewritten.
>> We have modified the sentence in the revised manuscript (lines 112-115).

4. Results, line 118 and onwards, I am slightly conflicted with this statement. The authors claim that if a particular protein is a receptor for a specific ligand, the loss of function mutant should not display any responses upon elicitation with that ligand. While this is primarily shown for ligands in the form of small peptides, where specific amino acids sequence is recognized and bound by the ligand-perceiving receptor, I would be more cautious with one-

to-one translating this trend to all types of ligands (hormones, lipids, pectin-derived oligogalacturonides (OGs)). It could be very much that there are different biologically active species of ligand molecules that can have partial affinity towards either, as mentioned by the authors, bona fide receptors or some homologs.
>> We thank the reviewer for this comment. We have now included examples of peptide, glycan and small molecule receptors, whose single deletion results in complete insensitivity to the respective ligand (lines 118-124). In certain cases, knock-out of closely related paralogs (*e.g.* PEPR1/2 for AtPep peptides, or P2K1/2 for eATP) (Pham et al., 2020, https://doi.org/10.1104/pp.19.01265; Yamaguchi et al., 2010, https://doi.org/10.1105/tpc.109.068874) was needed to observe full insensitivity. Therefore, we think that our hypothesis that knock-out mutation of a single WAK or closely related WAKs as proposed OG receptor(s) would lead to full insensitivity to OGs is valid.

5. Results, line 125 and onwards -Could this be driven by growing plants in two different photoperiods? The data presented in this manuscript were obtained in the short-day conditions of 10 hours of light and 14 hours of darkness, and the authors of Kohorn et al., 2021 used the long-day photoperiods of 16 hours of light and 8 hours of darkness.
>> This is an interesting thought. The difference in ROS production in response to OGs (and flg22) in the *wakΔ* and *wakΔ2* mutant could be indeed because of different growth conditions, soil or fertilizer used. However, as we do see clear OG-induced responses under our short-day conditions, the OG receptor(s) is/are likely to be expressed under the plant cultivation conditions used in our study. We mention in the discussion that, in our opinion, the differences for the *wak* mutants observed under short- and long-day conditions might further underline the role of WAKs as accessory RKs under certain growth conditions. This however does not change the major conclusion of the study that WAKs are genetically dispensable for OG-induced responses.

6. I was wondering if the development of the wakΔ2 mutant line was undistinguishable from wild-type plants through the entire life cycle. In line with this, in Figure 3C, I would like to see the absolute fresh weight of the seedlings grown in the mock conditions.
>> We thank the reviewer for the comment. Under our growth conditions, the deletion of the *WAK* gene cluster does not affect plant growth during the entire life cycle (see Rebuttal Figure A below). This is in line with studies on an independent *wak-quintuple* mutant (Lui *et al.*, 2023; 10.1126/sciadv.adf7714) and on the *wakΔ* mutant (Kohorn *et al.*, 2021; https://doi.org/10.1371/journal.pone.0251922), which reported wild-type phenotypes for these *wak* mutants in term of overall growth and development. Due to journal restrictions, we would like to suggest to not include the images of flowering plants into the revised manuscript.
As suggested by the reviewer, we replaced the relative seedling growth inhibition by absolute seedling fresh weight in Figure 3.

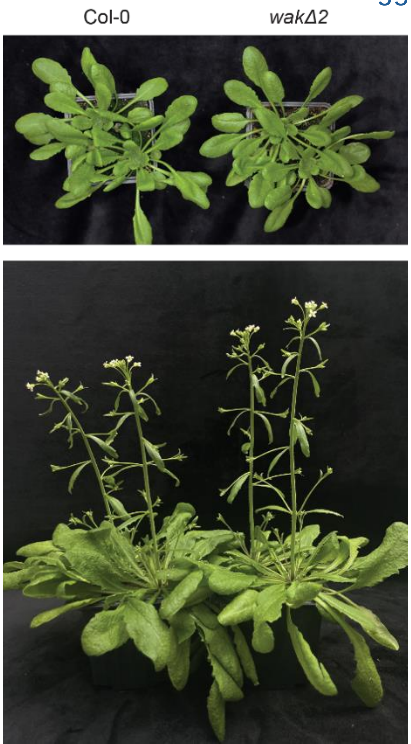

Rebuttal Figure A: Pictures of six-week-old (top) and of eight-week-old (bottom) Col-0 and *wakΔ2* mutant plants.

7. Figure 2- What do the authors think about the primed MAPK activity in the wakΔ2 mutant line upon OG3 treatment at 0 minutes? Is 0 minutes corresponding to mock-only treatment or just immersing the seedlings in 100 μg/mL OG10-15 or 100 μg/mL OG3? If the latest one, mock treatment is missing.
>> We thank the reviewer for pointing this out. We have repeated all MAPK assays in triplicates including mock controls and replaced Figures 2C and 2D with the new results including mock controls. This indicates that there is no primed MAPK activation in seedlings of the *wakΔ2* mutant.

8. Results, line 143 and onwards. While in plant pathology, callose deposition is typically viewed after hours, it is also an early response to MAMP challenge or pathogen infection that occurs within minutes. I suggest reviewing recent works by the plasmodesma community for more information and references.
>> We thank the reviewer to point this out. While callose deposition might be induced within minutes in the context of immune signaling within plasmodesmata, it is a well-accepted later response in the context of general immune responses triggered by many elicitors (Yu et al., 2017, https://doi.org/10.1146/annurev-phyto-080516-035649), which is what we have measured here in the context of OG-induced responses.

9. What do the authors think about the enhanced expression of PR1 in wakΔ2 mutant upon OG10-15 compared to Col-0? The results section states, "Both flg22 and OG10-15 -induced PR1 accumulation was not affected in wakΔ2 (FIGURE 3D). OG3 induced very weak PR1 accumulation; however, no difference in PR1 accumulation could be detected between Col-0 and wakΔ2 (FIGURE 3D)." Is that the case that no difference in PR1 accumulation in the wak2 versus Col was detected?
>> We thank the reviewer for this comment and agree that OG-induced PR1 activation might be weaker for Col-0 compared to the *wakΔ2* mutant in the replicate shown. However, this trend was not consistent in the other full factorial replicates. To avoid any potential misinterpretation, we exchanged the blot in Figure 3D with another one that is indeed more representative of the results obtained in different independent experiments.

10. As much as I am impressed by the clear and high-quality data that the authors presented in this manuscript falling into one coherent story demonstrating that the CRISPR cas quintuple does not display any altered defence response upon OGs or other elicitors induction in comparison to Columbia O plants... It makes me wonder how peculiar these observations are. Removing the entire WAK cluster makes (keeps?) plants indistinguishable from Col-0 when checking on different developmental stages and using various methods to monitor broadly understood PTI responses. I would like to ask the authors to consider the following
- demonstrating the sequencing results demonstrating that, indeed, the entire cluster of 5 WAK members is not present in the wakΔ2 mutant line
>> We thank the reviewer for this comment. Independent from the study presented here it has already been shown that the deletion or mutation of *WAKs* has no strong impact on plant growth (Lui *et al*., 2023; 10.1126/sciadv.adf7714, Kohorn *et al*., 2021; https://doi.org/10.1371/journal.pone.0251922). Additionally, Kohorn *et al*. (2021) showed wild type-like transcriptional responses of marker genes upon OG treatment in the *wakΔ* mutant.

We added the chromatogram of the Sanger sequencing to Figure 1 of the revised manuscript.

- the quintuple lines of (quintuple1 and quintuple2) were also described in in Lui et al., 2023. Would it be possible to validate some of the results obtained for wakΔ2 using the before-mentioned mutant lines to validate some of the obtained results?
>> We thank the reviewer for this suggestion. We have tested MAPK activation and PR1 accumulation in the *wak-quintuple 1* (*wakq-1*) mutant upon treatment with flg22, OG_3, and OG_{10-15} (Rebuttal Figure B). For both elicitor-induced MAPK activation and PR1 accumulation, the *wakq-1* and *wakΔ2* mutants behave similarly. To keep this manuscript as simple as possible, we would prefer not to include these data into the revised manuscript.

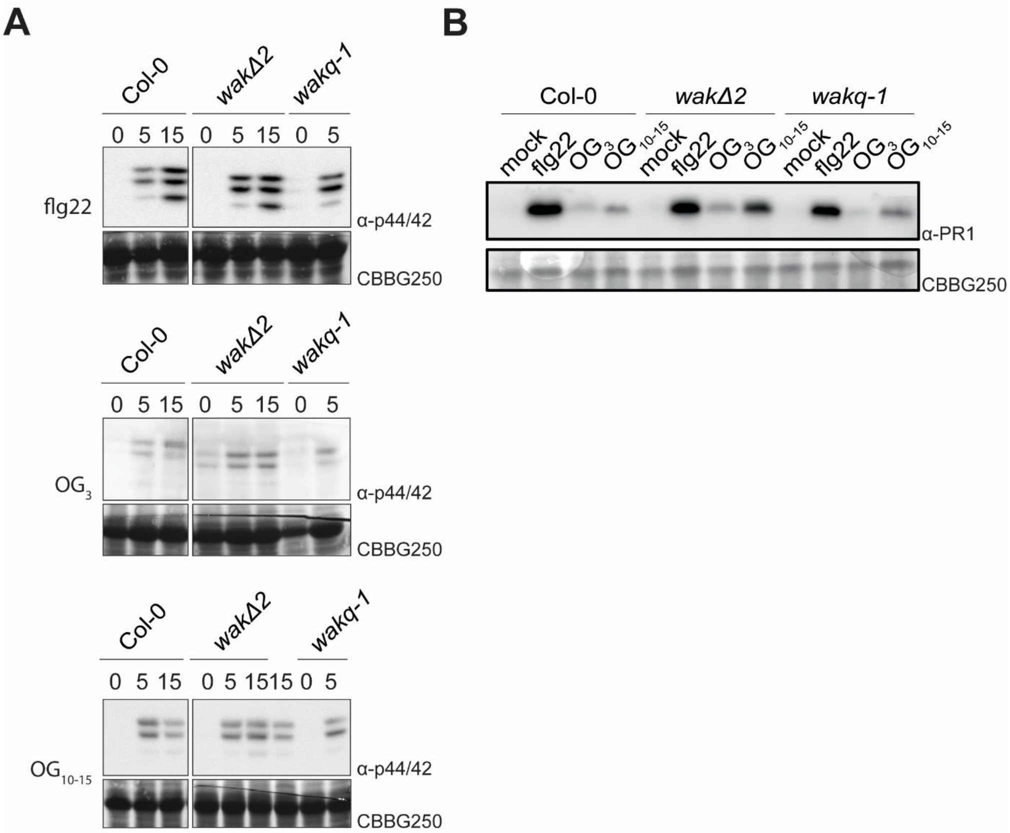

Rebuttal Figure B: MAPK activation in two-week old (A) and PR1 accumulation in three-week old (B) Col-0, *wakΔ2* and *wakq-1* mutant plants.

11. The authors of this paper presented that in the wakΔ2 defence responses are not changed compared to the Col-0 control. This makes me immediately curious: What, then, is being changed? Does the absence of WAK cluster genes trigger any compensation mechanisms? Are the WAKL genes undergoing any transcriptional changes? I was wondering if it would be possible to provide transcriptional data for wakΔ2 versus Col-0
>> We thank the reviewer for this suggestion. To address this point in an unbiased manner, we have now performed a whole-genome RNA-seq experiment comparing Col-0 and *wakΔ2* seedlings (see Rebuttal Figure C).
This analysis first showed that, in the *wakΔ2* mutant, no reads were detected for *WAKs* except for *WAK4*, for which approx. 600 nt of the 5' UTR are still encoded; thus, independently confirming that *wakΔ2* is a true loss of function mutant for the five Arabidopsis *WAK* genes.
Surprisingly, only three genes were significantly differentially expressed between Col-0 and *wakΔ2*: *WAK1*, *WAK2* and *miR850*, in line with the targeting of these genes and the very low expression of *WAK3*, *4* and *5* under these conditions. This indicates that *WAKs* are unlikely

to be involved in basic developmental processes (at least in unchallenged seedlings). These results are in line with previously published RNA-seq data of the *wakΔ* mutant (Kohorn *et al.*, 2021; https://doi.org/10.1371/journal.pone.0251922), showing no major transcriptional changes except for the deleted *WAKs*.
Finally, the expression of *WAKLs* was unaltered in the *wakΔ2* mutant, indicating that there is no compensatory mechanism due to the loss of *WAKs*.

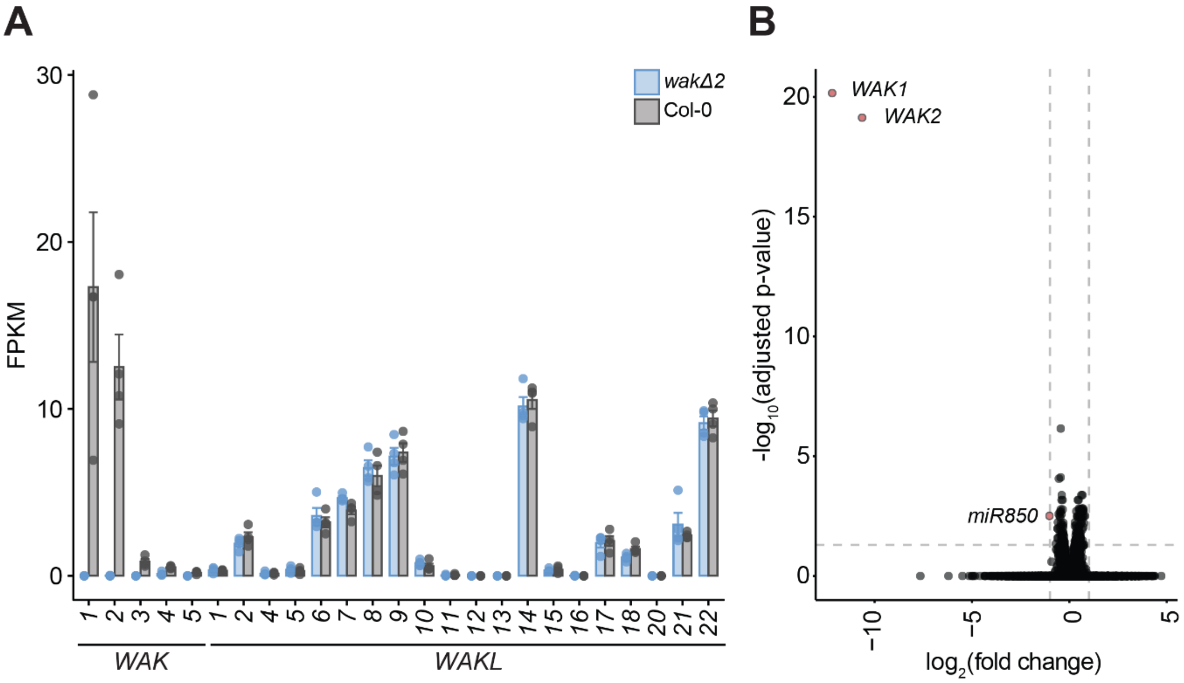

Rebuttal Figure C: Summary of RNA-seq experiment comparing two-week-old liquid grown Col-0 and *wakΔ2* seedlings. A. Detailed analysis of *WAK* and *WAK-like* transcript levels. B. Overall changes in Col-0 vs. *wakΔ2* transcriptomes.

12. General comment 1: The authors use plants at different developmental stages for different experimental approaches. It would be valuable for the reader if this were stated more explicitly.
>> We thank the reviewer for this comment. We have now clearly stated plant stages in the figure legends.

13. General comment 2: For most, if not for all, the figures, it seems completely senseless to present and annotate individual biological/technical repeats. It is not possible to distinguish them on the box plots anyway, and addicting this additional information to the figure makes it very difficult to read.
>> We thank the reviewer for this comment. We would like to keep the annotation of individual biological replicates to depict transparently data from multiple independent experiments, as it is commonly done (and encouraged) in many journals.
Yet, to improve the comprehensibility of the figures, we have now removed biological replicates from figure legends and instead mention in the figure captions that "shapes indicate biological/technical replicates".

14. General comment 3: The arrangement of the figure panels seems disproportional for some figures, like Figure 2 and Figure 4.
>> We thank the reviewer for this comment. We have revised all figures and made size panels more homogenous.

Response to Reviewer #2

This manuscript by Herold et al. presents a provocative finding that challenges the accepted role of Arabidopsis WALL-ASSOCIATED KINASES (WAKs) in perceiving oligogalacturonides (OGs) and initiating immune responses. The study uses genetic evidence to demonstrate that WAKs are not essential for OG-induced signalling and immunity in Arabidopsis, suggesting that WAKs have a different function and, potentially, that other receptors might be involved in this process. This finding challenges the well-accepted notion in the field that WAKs are OG receptors, and therefore their claims must be carefully presented.

One of the critical gaps in the presented data is the source and quality control of the OGs used in the experiments. The authors rely on a single OG commercial source without an explicit quality control, which might lead to misleading conclusions if the OG preparations are contaminated or functionally inconsistent. I suggest incorporating a quality check (e.g. GC/MS, HPLC/MS, HP-SEC-MS) to confirm that these preparations contain only OG. Additionally, employing a secondary source of OGs, whether commercial or derived from controlled digestion of homogalacturonan, would provide a robust foundation to validate the observed responses.
>> We thank the reviewer for this comment. We have mainly used commercial OG_{10-15} from Elicityl (GAT114). However, as shown in Rebuttal Figure D (western blots from same experiment as in Rebuttal Figure B), we have tested MAPK activation (A) and PR1 accumulation (B) in the *wakΔ2* mutant (and an additional *wak-quintuple* mutant *(wakq-1,* Lui *et al*., 2023; 10.1126/sciadv.adf7714*)* additionally upon treatment with OGs enriched in a degree of polymerization 9-15 described in Kohorn *et al*., (2014, https://doi.org/10.1074/jbc.M114.567545). We could not detect any differential MAPK activation or PR1 accumulation in Col-0 and the *wakΔ2* mutant upon treatment with the two OG preparations (see Rebuttal Figure D). Furthermore, treatment of the *wakΔ2* mutant with the homogenous elicitor GalA3/OG_3 resulted in wild type-like MAPK activation and PR1 accumulation, indicating that *WAKs* are not required for the perception of OGs derived from different sources and with different degrees of polymerization, in line with the general conclusion of our manuscript.

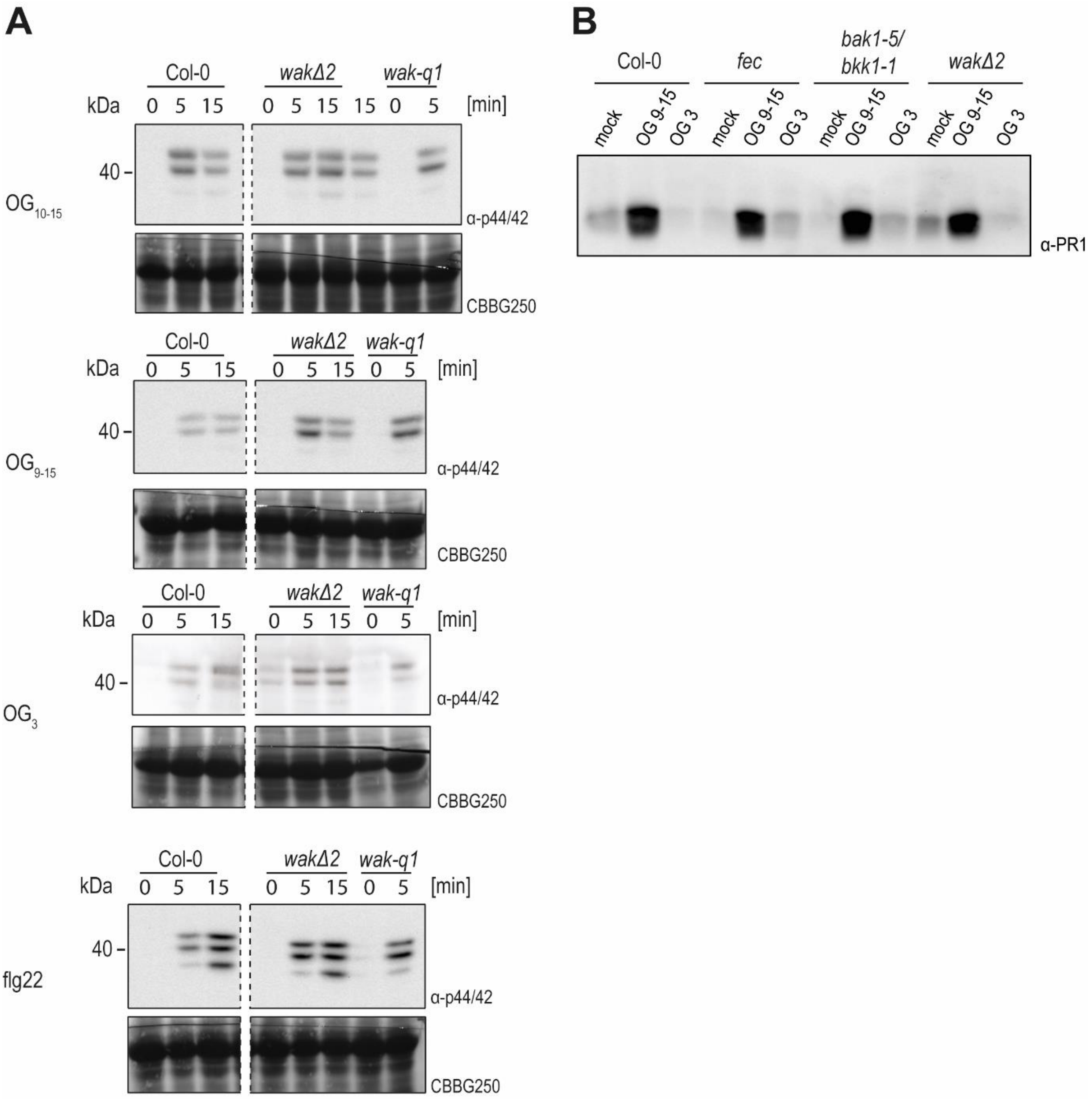

Rebuttal Figure D: MAP kinase activation (A) and PR1 accumulation (B) in the genotypes indicated in response to different OGs and flg22.

Another missing element in the experimental setup is the measurement of a PTI that I am sure the authors are familiar with: Ca2+ influx. This is a well-documented early signalling event in plant immune responses triggered by different MAMPs/DAMPs including OGs. I suggest including this in Figure 2, in order to provide a more comprehensive view of the signalling cascades involved.

>> We thank the reviewer for this suggestion. However, we do not have the genetic material to measure OG-induced Ca^{2+} influx in the *wakΔ2* background. While the addition of OG-induced Ca^{2+} data would provide an additional layer in early immune signaling, we have already reported a wide array of early and late PTI responses. The conclusion that WAKs are not required for OG-induced responses is because no difference could be observed between Col-0 and *wakΔ2* in these assays. Were Ca^{2+} influx be affected, it would still no fit with a proposed role of WAKs as OG receptors, as loss-of-function mutations in ligand-binding receptors would equally affect all downstream responses.

The western blots presented in the manuscript to analyse early and late PTI display several

inconsistencies and technical issues that could affect the interpretation of the results. Figure 2C includes BAK1 as a loading control, but not 2D (despite the legend indicating that it was used in both). For the OG 10-15 treatment in the wakΔ2 mutant, there is a band possibly for MAPK4-11, which is not usual for OGs. For OG3 in the wakΔ2 mutant it seems to be already some activation at t0. In Figure 3D, there is a surprising and interesting stronger activation in the wakΔ2 mutant in comparison to the wild type, however the text says that no difference could be detected (line 157 - 158).

>> We thank the reviewer for this comment. In line with a comment from another reviewer, we have repeated all MAPK assays in triplicates including mock controls and replaced Figures 2C and 2D with new blots including mock controls. There is no primed MAPK activation in seedlings of the *wakΔ2* mutant. In our hands, $OG_{10\text{-}15}$ induces weak activation of MPK4/11. This is however dependent on the exposure time and substrate used for detection of the chemiluminescence. While phosphorylated MPK4/11 was not detected using Pierce ECL Western Blotting Substrate (Thermo Fisher Scientific), it was detected using SuperSignal West Femto substrate (Thermo Fisher Scientific; see Rebuttal Figure E below). This points towards a weak phosphorylation of MPK4/11 upon treatment with different OG preparations, which is also consistent with a previous study by Gravino *et al*. (2017, https://doi.org/10.1111/mpp.12419).

For the PR1 blot, we agree with the reviewer that OG-induced PR1 activation might be weaker for Col-0 compared to the *wakΔ2* mutant in the replicate shown. However, this trend was not consistent in the other full factorial replicates. To avoid any potential misinterpretation, we exchanged the blot in Figure 3D with another one that is indeed more representative of the results obtained in different independent experiments.

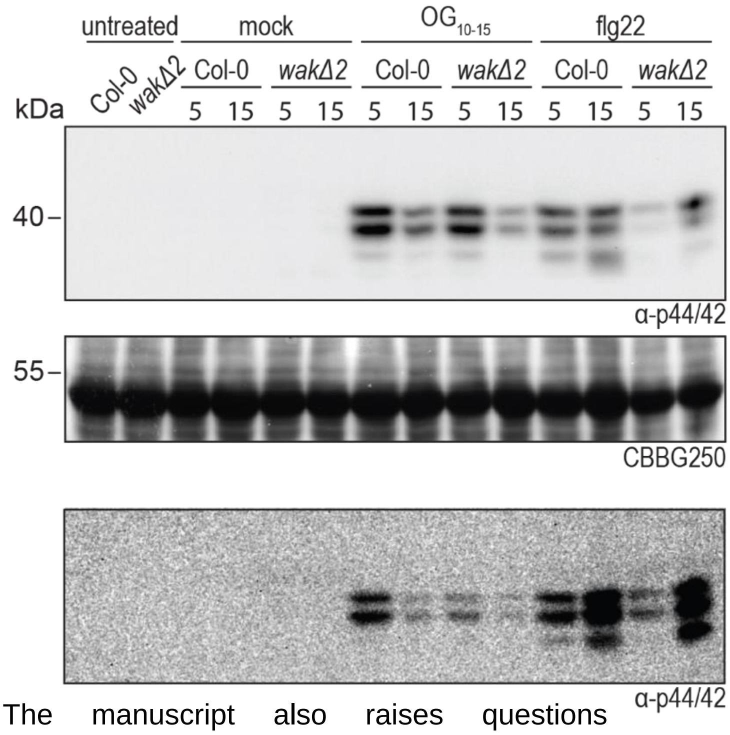

Rebuttal Figure E: MAP kinase activation in Col-0 and *wakΔ2* in response to the treatment indicated. Top, detection using SuperSignal West Femto substrate; bottom, detection using Pierce ECL Western Blotting Substrate.

The manuscript also raises questions regarding the comparative analysis of different mutants. The observation that a partial deletion mutant has a stronger phenotype (at least in terms of ROS production) than a complete delection is intriguing. I wonder why the authors did not include wakΔ (the partial deletion) as a control/comparison.

>> We thank the reviewer for this comment. We have tested OG-induced MAPK activation, ROS production, ethylene biosynthesis, seedling growth inhibition and induced resistance to *Pseudomonas syringae* pv. *tomato* also for the *wakΔ* mutant (Rebuttal Figure F). In our hands and growth conditions, the *wakΔ* mutant behaves like Col-0 and the *wakΔ2* mutant. The difference in ROS production in response to OGs (and flg22) observed by Kohorn et al

(2021, https://doi.org/10.1371/journal.pone.0251922) for the *wakΔ* mutant could be due to different growth conditions, soil or fertilizer used. This however does not change the major conclusion of the study that WAKs are genetically dispensable for OG-induced responses.

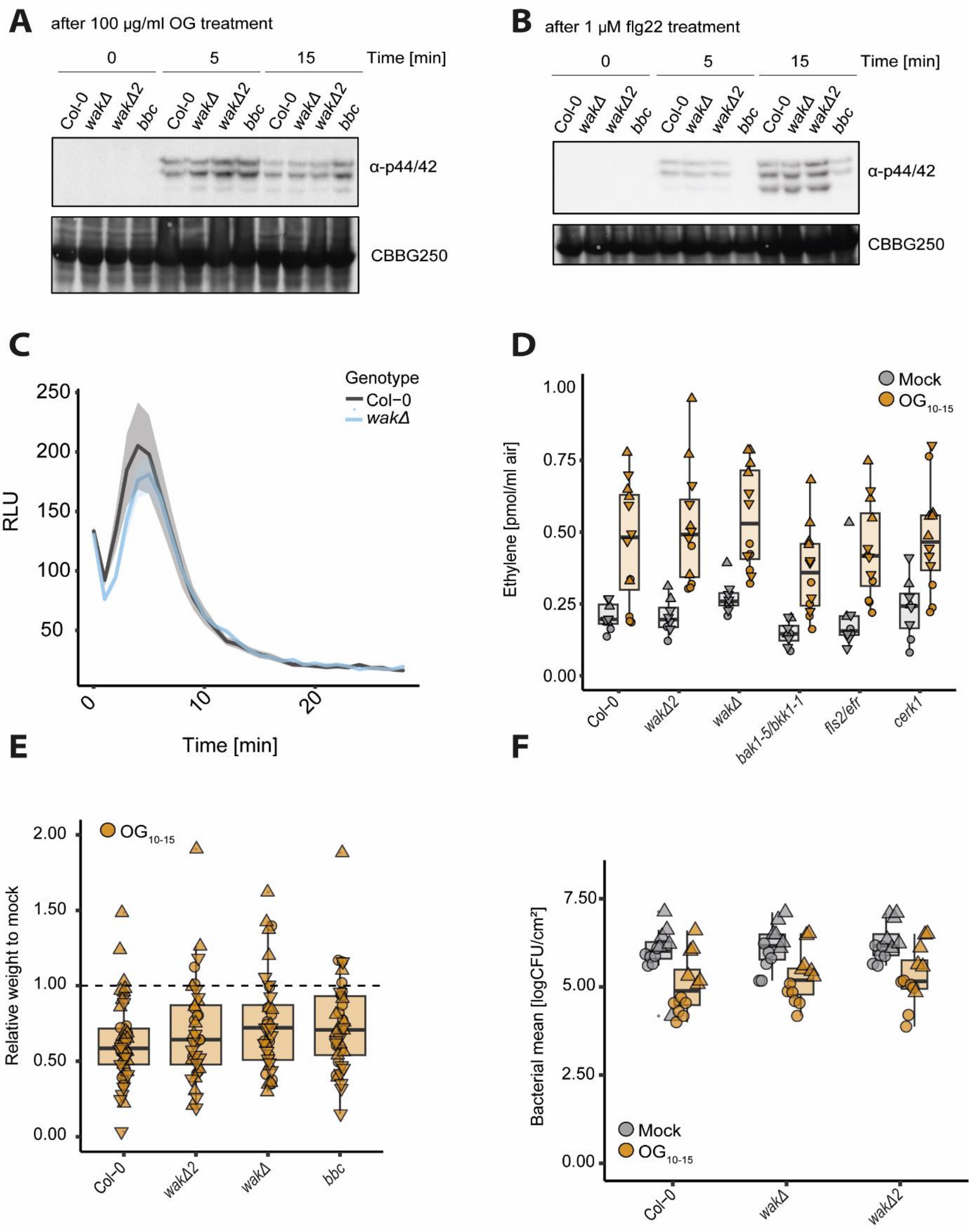

Rebuttal Figure F: Comparison of Col-0, *wakΔ2* and *wakΔ* in response to OG (and flg22) as determined by MAP kinase activation (A,B), ROS production (C), ethylene biosynthesis (D), seedling growth inhibition (E), and induced resistance to *Pto* DC3000 (F).

Regarding ROS, in Figure 2A there seems to be a difference in the peaks of the two genotypes, although the effect is somehow buffered in the total RLU measurements (Figure

2B). Perhaps it is a difference that the authors should at least acknowledge. Furthermore, the absence of observable phenotypes in mutants with no WAK expression contradicts earlier studies using antisense RNA where such modifications resulted in lethality or clear phenotypic changes. This contradiction should be addressed, possibly suggesting alternative explanations or experimental limitations that could account for these differences.
>> We thank the reviewer for this suggestion. ROS production experiments can be quite variable and the difference in the two genotypes is in accordance with the variations typically observed for ROS production experiments in our laboratory.
While previous studies using antisense RNA to target the *WAKs* resulted in lethality or clear phenotypic changes, three CRISPR/Cas9 *WAK* deletion mutants, including the *wakΔ2* mutant presented here, show no pronounced phenotypic changes (Kohorn *et al*., 2021, https://doi.org/10.1371/journal.pone.0251922, Liu *et al*., 2023, 10.1126/sciadv.adf7714). Therefore, we assume that the severity of the developmental phenotypes previously observed using antisense RNA might be due to cross-silencing of other genes (putatively *WAKLs*) or an artefact of the approaches used.
As indicated in response to Reviewer 1, we have now performed an RNA-seq experiment comparing Col-0 and *wakΔ2* seedlings and found that only three genes are differentially expressed in Col-0 and *wakΔ2* mutant seedlings: *WAK1*, *WAK2* and *miR850* (see Rebuttal Figure C). This indicates that *WAKs* are unlikely to be involved in basic developmental processes (at least in unchallenged seedlings).

Minor: some references are missing from the reference list, for example these relative to FERONIA (Feng et al 2018, Tang et al 2022, Lin et al 2022).
>> We thank the reviewer for this comment. We went through all references again and added those three references to the reference list (lines 493, 576, 634).

In any case, the manuscript is interesting and well-written, and the results could potentially open new avenues for research into the real receptors of OGs and the biological relevance of WAKs. However, these findings must be supported by rigorous experimental validation to gain wider acceptance in the field.

Response to Reviewer #3

The paper by Herold et al. is of major interest as it revolutionizes the current textbook model describing WAK receptors as GalA10-GalA15 receptors. While I have no comments on the experiments performed, I do have comments regarding the way the paper describes and names the OGs they tested.

The authors specify that oligogalacturonides are 'pectin-derived oligogalacturonides.' For their experiments, the 'authors' specifically use to a mixture of oligogalacturonides ranging from DP10 to DP15, produced from polygalacturonic acid, i.e., from chemically fully demethylesterified pectins. Since they discuss the fact that endogenous OGs detected in vivo display chemical decorations such as methylesterification and/or acetylation (l. 230-231) and that these methyl- and acetylated OGs could be WAK substrates, it should be clear both in the text and in the title to which OGs the authors are referring, i.e., deca to pentadeca fully demethylesterified OGs as it is done line 228. Therefore, I would recommend using GalA10-GalA15 instead of OG10-15 to avoid any confusion as it has been done on line 83 for GalA3.
>> We thank the reviewer for this comment. We agree that that is important to state clearly which OGs were used. However, in all publications about WAKs and OGs, the authors refer to OG_{10-15}. Therefore, to prevent confusion, we would like to stick to OG_{10-15}. To keep it consistent, we however changed $GalA_3$ to OG_3.

Other comment :
Line 45. OGs are generated from demethylesterified pectins.

It's not accurate. When digested with a pectin lyase, methylesterified OG are produced from highly methylesterified pectins.

>> We thank the reviewer for this comment. We have rephrased this section to be more precise (lines 44-46).

Dear Cyril,

We have received reviews of your manuscript entitled "Arabidopsis WALL-ASSOCIATED KINASES are not required for oligogalacturonide-induced signaling and immunity." On the basis of the advice received, the board of reviewing editors would like to accept your manuscript for publication in The Plant Cell. This acceptance is contingent on revision based on the comments of our reviewers. In particular, please consider the following:

All reviewers flag some small points for improvement, which we ask you to address them in the final revision. While we do not request new experimental data, the reviewers made clear statements about the value of including data that is now included only in the response to reviewers. We ask you to do so accordingly.

Please highlight all changes and include a detailed annotation of changes of the text, with line numbers, and noting your responses to the comments.

To submit your revised manuscript, click:

Link Not Available

If you have any questions about the revision submission procedures, please contact the Editorial Office Staff (tpc-submissions@aspb.org). If you cannot return the revised manuscript within 30 days, please let us know. Otherwise, we will assume that you have elected not to revise the manuscript and withdraw it.

Thank you very much for the privilege of reviewing this work. I look forward to receiving the next version.

Sincerely,

The Plant Cell Editorial Board

(Please note that due to a potential conflict of interest, EIC Blake Meyers did not participate in evaluating this manuscript).

Reviewer #1 (Comments for the Author):

I read the revised version of the manuscript"Arabidopsis WALL-ASSOCIATED KINASES are not required for oligogalacturonide-induced signaling and immunity" by Herold et al. with great pleasure. I want to thank the authors for their dedication to cautious and thoughtful responses to my and other reviewers' comments. I am satisfied with the responses in general. I do have, however, two minor remarks:

1- I have a slight reservation about the responses provided regarding my comments shared in points 7 or 9, as they suggest that the most representative results were not always shown in the initial submission. Or the conclusions were not always supported by the data or their representation. This is pretty much fixed now.
2- I appreciate the RNAseq data, but a- done at a different development stage (2 weeks-old seedlings grown in the liquid culture) than most of the other immunity-related results b- it is still a question of other WAKL will not start compensating upon OGs induction.

Reviewer #2 (Comments for the Author):

The manuscript "Arabidopsis WALL-ASSOCIATED KINASES are not required for oligogalacturonide-induced signaling and immunity" has undergone thorough review, revealing a study that challenges our current understanding of WAKs as OG receptors in Arabidopsis. The authors have made substantial improvements in response to the initial review, strengthening their case with additional experiments and clarifications. The authors have addressed most of my questions satisfactorily. While their statement that "loss-of-function mutations in ligand-binding receptors would equally affect all downstream responses" could be subject to further discussion, I acknowledge that generating the genetic material for Ca2+ measurements might unnecessarily delay the publication of this manuscript. Given the overall strength of the evidence presented, I believe this particular point does not significantly impact the main conclusions of the study and therefore does not warrant further delay in publication.
Apart from that, the authors have conducted comprehensive comparisons of immune responses across Col-0, wakΔ2, and wakΔ mutants, providing a more robust foundation for their conclusions. Their RNA-seq analysis of Col-0 and wakΔ2 seedlings offers valuable insights into the genetic consequences of WAK deletion. The manuscript now presents a clearer picture of the experimental conditions, with improved figures that effectively communicate their findings. There are only few minor comments:
- Line 52: The pattern-triggered immunity (PTI) abbreviation is introduced in the abstract but not again in the main body of the manuscript, unlike other abbreviations which are defined in both the abstract and main text.
- The CRISPR-Cas9 mutagenesis section lacks information on strategies employed to avoid or evaluate potential off-target

effects -if any.

While the manuscript is nearly ready for publication, it would benefit significantly from the inclusion of the RNA-seq data, at least as supplementary material. This would give a genome-wide perspective on the effects of WAK deletion, supporting the authors' claim that WAKs are not central to OG-induced signaling. Besides, the unexpected -at least, to me- differential expression of miR850 raises intriguing questions about either secondary consequences of WAK deletion or potential off-target effects. Including this data would allow readers to assess this finding independently and potentially spark new research directions. Besides, presenting the full dataset would enhance the reproducibility and transparency of the study, allowing other researchers to build upon this work.

With this addition and the suggested minor revisions, I believe this study will make a significant contribution to our understanding of plant immune signaling and stimulate new research directions in the cell wall-mediated immunity field.

Reviewer #3 (Comments for the Author):

Concerning Rebuttal Figure D, the authors claim in their response to the reviewers that 'treatment of the wakΔ2 mutant with the homogeneous elicitor GalA3/OG3 resulted in wild-type-like PR1 accumulation.' However, what I observe is that OG3 seems to inhibit PR1 accumulation rather than promote it. I agree that this not consistent with what the authors show in Rebuttal Figure B. Therefore, I would not recomment to include GalA3 results to the paper.

In addition, the fact that the authors observed similar MAPK activation using the mutant from Liu et al. suggests to me that the conclusion should instead be that WAKs are genetically dispensable for OG-induced responses under short-day conditions. I would also recommend including at least one experiment using plantlets grown under long-day conditions to test PR1 activation in their mutant following OG10-15 treatment

Please also note the following:

-The Plant Cell now requires authors to complete and submit an author revisions checklist upon submission of a revised manuscript. The aim of the checklist is to aid authors in preparing a high-quality manuscript, facilitate the review and assessment of revised manuscripts, and help to ensure that journal standards are maintained across the board. If your manuscript is accepted, the completed checklist will be published as supplemental material attached to the article online. Please download a copy of the checklist (pdf fillable form) at this link, for submission with your revised manuscript: https://tpc.msubmit.net/html/Author_Revisions_Checklist.pdf.

-Supplemental materials should be restricted to large datasets and tables, presentation of replicates, and validation of reagents, methods, or genotypes. Any data that are used to support the major claims must be in the main manuscript. Supplemental figure legends must indicate what figure in the main manuscript is supported by the supplemental data presented. Please justify how each of the supplemental figures meet the criteria.

-Sampling methods and the nature of "biological replicates" should be described precisely (i.e. different plants, parts of plants, pooled tissue, independent pools of tissue, sampled at different times, etc.), along with a clear description of and rationale for any statistical analyses conducted. The reader should know exactly what was sampled; what forms the basis of the calculation of any means and statistical parameters reported. This is also necessary to ensure that proper statistical analysis was conducted.

Dear Editors,

Thank you for your positive decision and the comments received from the three reviewers.
We have now addressed them as noted below in our point-by-point response.
We therefore hope that our manuscript can now formally be accepted for publication in The Plant Cell.

Best regards,
Cyril Zipfel, on behalf of all authors.

<u>Response to Reviewer #1</u>

I read the revised version of the manuscript"Arabidopsis WALL-ASSOCIATED KINASES are not required for oligogalacturonide-induced signaling and immunity" by Herold et al. with great pleasure. I want to thank the authors for their dedication to cautious and thoughtful responses to my and other reviewers' comments. I am satisfied with the responses in general.
>> Thank you very much for the appreciation of the efforts we have made to address previous comments.

I do have, however, two minor remarks:

1- I have a slight reservation about the responses provided regarding my comments shared in points 7 or 9, as they suggest that the most representative results were not always shown in the initial submission. Or the conclusions were not always supported by the data or their representation. This is pretty much fixed now.
>> As mentioned in our previous revision, we have performed additional experiments, which have been added to the manuscript to strengthen our conclusions.

2- I appreciate the RNAseq data, but a- done at a different development stage (2 weeks-old seedlings grown in the liquid culture) than most of the other immunity-related results b- it is still a question of other WAKL will not start compensating upon OGs induction.
>> Our RNA-seq experiments were performed using 2-week-old seedlings, which is the same plant stage used to evaluate OG responsiveness as measured using MAP kinase activation and seedlings growth inhibition assays (Figs. 2C-D, 3C).
While we cannot completely exclude the possibility that some WAKLs might be differentially expressed upon OG perception, their induction upon this initial signaling activation in a mutant knock-out for all *WAK* genes would still indicate that WAKs are <u>genetically dispensable</u> for OG perception and ensuing signaling – which is the main message of our manuscript.

<u>Response to Reviewer #2</u>

The manuscript "Arabidopsis WALL-ASSOCIATED KINASES are not required for oligogalacturonide-induced signaling and immunity" has undergone thorough review,

revealing a study that challenges our current understanding of WAKs as OG receptors in Arabidopsis. The authors have made substantial improvements in response to the initial review, strengthening their case with additional experiments and clarifications. The authors have addressed most of my questions satisfactorily. While their statement that "loss-of-function mutations in ligand-binding receptors would equally affect all downstream responses" could be subject to further discussion, I acknowledge that generating the genetic material for Ca2+ measurements might unnecessarily delay the publication of this manuscript. Given the overall strength of the evidence presented, I believe this particular point does not significantly impact the main conclusions of the study and therefore does not warrant further delay in publication.
Apart from that, the authors have conducted comprehensive comparisons of immune responses across Col-0, wakΔ2, and wakΔ mutants, providing a more robust foundation for their conclusions. Their RNA-seq analysis of Col-0 and wakΔ2 seedlings offers valuable insights into the genetic consequences of WAK deletion. The manuscript now presents a clearer picture of the experimental conditions, with improved figures that effectively communicate their findings.
>> Thank you very much for the appreciation of the efforts we have made to address previous comments.

There are only few minor comments:

- Line 52: The pattern-triggered immunity (PTI) abbreviation is introduced in the abstract but not again in the main body of the manuscript, unlike other abbreviations which are defined in both the abstract and main text.
>> We have now addressed this.

- The CRISPR-Cas9 mutagenesis section lacks information on strategies employed to avoid or evaluate potential off-target effects -if any.
>> BLAST searches against 'Araport11 genomic locus sequences (DNA)' show that the oligonucleotides used as templates for SgRNA-targeted sites only result in high-scoring matches with *WAK4* and *WAK2*, respectively.

While the manuscript is nearly ready for publication, it would benefit significantly from the inclusion of the RNA-seq data, at least as supplementary material. This would give a genome-wide perspective on the effects of WAK deletion, supporting the authors' claim that WAKs are not central to OG-induced signaling. Besides, the unexpected -at least, to me- differential expression of miR850 raises intriguing questions about either secondary consequences of WAK deletion or potential off-target effects. Including this data would allow readers to assess this finding independently and potentially spark new research directions. Besides, presenting the full dataset would enhance the reproducibility and transparency of the study, allowing other researchers to build upon this work.
>> We are happy to add our RNA-seq data to the manuscript, and have added panels of previous Rebuttal Figure C to Figure 1 as panels E and F. We have also updated the methods section accordingly, and deposited the RNA-seq raw data to the European Nucleotide Archive under the accession number PRJEB80751.

Regarding *miR850*, it is important to note that while we mentioned it as being significantly differentially expressed between Col-0 and *wakΔ2*, it is barely so (fold-

change = -1.025561768), and it would be difficult to imagine how the slight down-regulation of this miRNA would affect our conclusion that WAKs are genetically dispensable for OG perception, which all our results (in this *wak* mutant alleles – but now also for other alleles provided as part of our previous rebuttal) otherwise indicate.

With this addition and the suggested minor revisions, I believe this study will make a significant contribution to our understanding of plant immune signaling and stimulate new research directions in the cell wall-mediated immunity field.

Response to Reviewer #3

Concerning Rebuttal Figure D, the authors claim in their response to the reviewers that 'treatment of the wakΔ2 mutant with the homogeneous elicitor GalA3/OG3 resulted in wild-type-like PR1 accumulation.' However, what I observe is that OG3 seems to inhibit PR1 accumulation rather than promote it. I agree that this not consistent with what the authors show in Rebuttal Figure B. Therefore, I would not recomment to include GalA3 results to the paper.
>> Thank you for acknowledging this point, based on our previous rebuttal.

In addition, the fact that the authors observed similar MAPK activation using the mutant from Liu et al. suggests to me that the conclusion should instead be that WAKs are genetically dispensable for OG-induced responses under short-day conditions. I would also recommend including at least one experiment using plantlets grown under long-day conditions to test PR1 activation in their mutant following OG10-15 treatment.
>> We thank the reviewer for this comment. While it might be interesting to analyse how responses to OGs differ under different light regimes, this is not the focus of this study. Short- vs long-day experiments would not contribute anything to receptor characterization. Putative receptor mutants either show OG deficiencies or not, but certainly not in a light-dependent manner.

30-Oct-2024

Dear Cyril,

We are pleased to inform you that your paper entitled "Arabidopsis WALL-ASSOCIATED KINASES are not required for oligogalacturonide-induced signaling and immunity" has been accepted for publication in The Plant Cell, pending a final editorial review by a science editor. At this stage, your manuscript will be evaluated by a science editor with respect to its presentation of scientific content, compliance with journal policies, and presentation for a broad readership. The Plant Cell has contracted with Plant Editors (planteditors.com) to provide this service to our authors, and you will soon receive additional information on this process.

Please note that each author needs to link their ORCID identifier to their account before your manuscript can be published. If any authors do not have an ORCID linked to their account, they will receive a message with a link to complete this task. Please follow up with your coauthors and ensure that they have completed this task as soon as possible.

The Plant Cell and The Arabidopsis Information Resource (TAIR) are collaborating to collect functional annotation data about Arabidopsis genes from authors. This includes information about the gene's molecular function (e.g., kinase activity, ATP synthetase activity), the biological process/es it is involved in (e.g., endosperm development, threonine biosynthesis), its subcellular location (e.g., nucleus, ER), anatomical or developmental expression pattern (e.g., leaf, ovule, flower stage 10, seedling stage), or its partner in a protein-protein interaction (e.g., AT1G01010 interacts with AT1G01020).

If your paper contains results falling into one or more of these categories for Arabidopsis genes, we request that you now submit these data for inclusion in TAIR by filling in the form provided at the following URL: https://www.arabidopsis.org/doc/submit/functional_annotation/123. If you need further clarification on what types of data can be submitted please contact curator@arabidopsis.org.

Finally, we encourage your submission of artwork for the journal cover. Monthly issues will have an online cover image and selected covers will be used for posters, other promotional items, and "wallpaper" for mobile devices. For more information, refer to Cover Submission in the Instructions for Authors https://tpc.msubmit.net/cgi-bin/main.plex?form_type=display_auth_instructions.

We look forward to seeing your paper published.

Sincerely

The Plant Cell Editorial Board

----FOR ASPB OFFICE USE ONLY (DO NOT EDIT)----
MSID: 39203
Corresponding Author: Prof. Dr. Zipfel
